# Effect of *DLK1* and *RTL1* but Not *MEG3* or *MEG8* on Muscle Gene Expression in Callipyge Lambs

**DOI:** 10.1371/journal.pone.0007399

**Published:** 2009-10-09

**Authors:** Jolena N. Fleming-Waddell, Gayla R. Olbricht, Tasia M. Taxis, Jason D. White, Tony Vuocolo, Bruce A. Craig, Ross L. Tellam, Mike K. Neary, Noelle E. Cockett, Christopher A. Bidwell

**Affiliations:** 1 Department of Animal Sciences, Purdue University, West Lafayette, Indiana, United States of America; 2 Department of Statistics, Purdue University, West Lafayette, Indiana, United States of America; 3 Animal Sciences Division, University of Missouri, Columbia, Missouri, United States of America; 4 School of Veterinary Science, The University of Melbourne, Parkville, Victoria, Australia; 5 CSIRO Livestock Industries, St. Lucia, Queensland, Australia; 6 Department of Animal, Dairy and Veterinary Sciences, Utah State University, Logan, Utah, United States of America; East Carolina University, United States of America

## Abstract

Callipyge sheep exhibit extreme postnatal muscle hypertrophy in the loin and hindquarters as a result of a single nucleotide polymorphism (SNP) in the imprinted *DLK1-DIO3* domain on ovine chromosome 18. The callipyge SNP up-regulates the expression of surrounding transcripts when inherited in *cis* without altering their allele-specific imprinting status. The callipyge phenotype exhibits polar overdominant inheritance since only paternal heterozygous animals have muscle hypertrophy. Two studies were conducted profiling gene expression in lamb muscles to determine the down-stream effects of over-expression of paternal allele-specific *DLK1* and *RTL1* as well as maternal allele-specific *MEG3, RTL1AS* and *MEG8*, using Affymetrix bovine expression arrays. A total of 375 transcripts were differentially expressed in callipyge muscle and 25 transcripts were subsequently validated by quantitative PCR. The muscle-specific expression patterns of most genes were similar to *DLK1* and included genes that are transcriptional repressors or affect feedback mechanisms in β-adrenergic and growth factor signaling pathways. One gene, phosphodiesterase 7A had an expression pattern similar to *RTL1* expression indicating a biological activity for *RTL1* in muscle. Only transcripts that localize to the *DLK1-DIO3* domain were affected by inheritance of a maternal callipyge allele. Callipyge sheep are a unique model to study over expression of both paternal allele-specific genes and maternal allele-specific non-coding RNA with an accessible and nonlethal phenotype. This study has identified a number of genes that are regulated by *DLK1* and *RTL1* expression and exert control on postnatal skeletal muscle growth. The genes identified in this model are primary candidates for naturally regulating postnatal muscle growth in all meat animal species, and may serve as targets to ameliorate muscle atrophy conditions including myopathic diseases and age-related sarcopenia.

## Introduction

Callipyge sheep are a well recognized model for muscle growth and polar overdominant inheritance. Callipyge animals exhibit a 30–40% increase in postnatal muscle growth of the pelvic and loin muscles as well as a 6–7% decrease in total fat content [1]–[5]. The muscle hypertrophy phenotype is the result of an increase in both the size and proportion of type IIB glycolytic myofibers, while the total number of myofibers is not affected [6]–[8].

**Figure 1:**
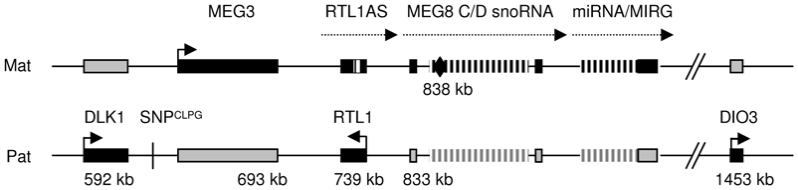
Organization of the *DLK1-DIO3* gene cluster in mammals. The relative positions of the maternal non-coding RNA genes and paternal protein coding genes are based on the bovine chromosome 21 sequence contig NW_001494068. The genes are shaded black on the parent-of-origin chromosome to indicate their allele-specific expression. The protein coding genes are expressed from the paternal allele (pat) and the non-coding RNA are expressed from the maternal allele (mat). The causative mutation (SNP^CLPG^) for the callipyge phenotype lies in the intergenic region between *DLK1* and *MEG3*. The black diamond indicates the approximate position of Affymetrix probe Bt18078.1.S1_at. The presence of C/D snoRNA and most miRNA have been detected in the bovine genome sequence but have not been confirmed in sheep.

The callipyge mutation (*C*) is a single nucleotide polymorphism that is located in an intergenic region between *Delta-like homologue 1 (DLK1)* and *Maternal Expressed Gene 3* (*MEG3*) in the *DLK1-DIO3* imprinted domain on ovine chromosome 18 (Figure 1) [9]–[11]. The callipyge phenotype was the first demonstration of polar overdominance in mammals [12], [13]. Paternal heterozygous (+/*C^Pat^*) lambs exhibit the callipyge phenotype of muscle hypertrophy, but maternal heterozygous (C*^Mat^*/+) and homozygous (*C/C*) lambs have muscle growth equivalent to normal (+/+) lambs [12], [13]. There has been evidence for polar overdominance effects of the orthologous *DLK1/MEG3* region on birth weight, weaning weight, and average daily gain in pigs [14], [15] and childhood obesity in humans [16].

The callipyge mutation has altered a putative long range control element [17] but has not altered the parent-of-origin allele-specific expression [10]. In all hypertrophied muscles of callipyge sheep (*+/C^Pat^*) that have been examined to date, there is elevated expression of both paternal allele-specific protein coding genes, *DLK1* and *Retrotransposon-like 1* (*RTL1*; Figure 1), making it ambiguous as to which gene causes muscle hypertrophy [10], [18]–[21]. Transgenic mice over-expressing *DLK1* in skeletal muscle had a significant increase in muscle mass and muscle fiber size compared to normal mice and this study concluded that *DLK1* was the cause of muscle hypertrophy in callipyge sheep [22]. Animals possessing a maternally inherited callipyge allele (*C^Mat^/+* and *C/C)* show up-regulation of the maternally expressed non-coding RNA transcripts (Figure 1): *RTL1-antisense (RTL1AS)*, *MEG3* and *Maternally Expressed Gene 8 (MEG8)* in skeletal muscle [10], [18], [20], [21], [23]. Homozygous (*C/C*) animals exhibit a *cis* effect of the mutation on both the paternal and maternal allele-specific genes, however, the paternal allele specific genes are expressed at a lower level than in callipyge (*+/C^Pat^*) lambs [10], [18]. This led to the hypothesis of a *trans*-interaction between the products of the maternal and paternal alleles to create the polar overdominant inheritance of the callipyge phenotype [17]. Four microRNA have been detected in *longissimus dorsi* of *C/C* animals from the maternal transcript, *RTL1AS*, along with their cleavage products from the paternal *RTL1* transcript, demonstrating a *trans*-interaction of the alleles in sheep [24]. This result suggests that *RTL1/RTL1AS* expression is responsible for polar overdominance and the biological activities of both genes contribute to muscle hypertrophy.

*DLK1* is widely expressed in many tissues during mammalian embryonic development [25]–[27] and is implicated as an important developmental growth factor. The glycoprotein has six EGF-like repeats in an extracellular domain and a transmembrane domain [28]. The circulating cleavage product was shown to be identical to fetal antigen 1, found in the serum of pregnant females and in amniotic fluid [28], [29]. Splice variants of *DLK1* have in-frame deletions of the sixth EGF repeat and parts of the juxtamembrane domain and those translation products remain membrane associated [30], [31]. A membrane bound splice variant of *DLK1* appears to be the predominant transcript in sheep muscle [22]. The DLK1 protein was highly abundant in the endomycium surrounding individual myofibers and the perimycium separating fiber bundles of hypertrophied callipyge muscles [8].

*RTL1*, also known as *PEG11*, has evolved from an ancestral *gypsy* LTR- retrotransposon insertion prior to the divergence of marsupials and placental mammals and has gained function in the mammalian genome [32], [33]. A placental function for the *RTL1* gene has been determined by gene targeting in mice to generate both *RTL1* knock-out and over-expression due to the loss of *RTL1AS* microRNA [34]. Both models showed developmental and growth changes related to the fetal-maternal interface of the placenta. Histological analysis showed abnormalities in the endothelial cells of the fetal capillaries at the maternal-fetal interface and the RTL1 protein was localized in the perinuclear areas of the endothelial cells [34].

All of the maternal allele-specific transcripts produce non-coding RNA. *MEG3* is expressed in many tissues during development including the central nervous system and most mesoderm derived tissues where it is co-expressed with *DLK1* [26]. Thirteen splice variants of *MEG3* exist in mouse embryos [35] and at least five mRNA variants have been detected in sheep skeletal muscle [36]. The *MEG8* gene is conserved between sheep and humans and four exons of the 5′ end of the *MEG8* gene were present in the ovine contig sequence (AF354168) [37]. One C/D snoRNA precursor was detected in an intron of the human and ovine genes and the human gene was predicted to host two additional clusters of C/D snoRNA precursors in the introns of *MEG8* (Fig. 1) [38]. The *Rian* gene (RNA imprinted and accumulating in nucleus) in the mouse was found in a similar location as *MEG8* and hosts nine C/D snoRNA but has little sequence similarity [38], [39].

The *DLK1*-*DIO3* domain contains the largest known microRNA cluster and has been detected in silico in all mammals with a complete genome sequence (Fig. 1) [40]–[43]. The microRNA genes were found as single genes and as tandem arrays in mouse and human [40], [41]. They are expressed from the maternal allele in the mouse embryo (head, body and placenta) but expression in the adult is restricted to the brain [41]. In the mouse, it has been suggested that there is a long maternal allele-specific transcript originating from *MEG3/Gtl2* gene through the microRNA containing gene (*Mirg*) [39] or there may be a series of overlapping transcripts [44]. The ovine genome sequence is not available for this region but many of these C/D snoRNA and microRNA genes are annotated in the bovine genome sequence and several of these microRNA have been detected by sequencing [45].

The purpose of this study was to identify downstream targets and pathways responding to the over-expression of paternal allele-specific and maternal allele-specific genes due to the callipyge mutation. We have used the Affymetrix Bovine Expression Array due to the high degree of similarity of ovine and bovine gene sequences [46] and the availability of the bovine genome sequence and annotation. We were making the assumption that the microarray probes detect the orthologous ovine transcript and previous results indicate that this was the case [47], [48]. The effects of up-regulation of *DLK1* and *RTL1* were measured by comparing callipyge (*+/C^Pat^*) to wild-type (+/+) muscles at three ages during the initial development of muscle hypertrophy and one age after hypertrophy was well established. The effects of the maternal transcripts *MEG3*, *MEG8* and their associated small RNA on muscle gene expression were determined at a single age by comparing *C^Mat^/+* and *C/C* expression profiles to *+/C^Pat^* and +/+ expression profiles.

Two previous studies have been published on gene expression in the *longissimus dorsi* of callipyge sheep by our group and our collaborators [47], [48]. The current study extends our knowledge by profiling the *semimembranosus*, which has the largest magnitude of hypertrophy [2], [3], [5] and uses a more extensive postnatal developmental series. Differences in gene expression profiles between the two muscles could be expected due to different locations, functions, and fiber type proportions of the normal muscles, but transcripts that are directly influenced by *DLK1* or *RTL1* should have a similar response in these two hypertrophied muscles. A subset of the genes were tested for differential gene expression by quantitative PCR in two muscles that become hypertrophied, the *longissimus dorsi* and *semimembranosus*, and one muscle that does not hypertrophy, the *supraspinatus*, in a larger set of callipyge sheep with known phenotypic data. The spatial and temporal patterns of gene expression will allow us to discern some direct transcriptional responses to *DLK1* and *RTL1* from secondary and tertiary changes in expression due to the muscle hypertrophy.

## Results

**Figure 2:**
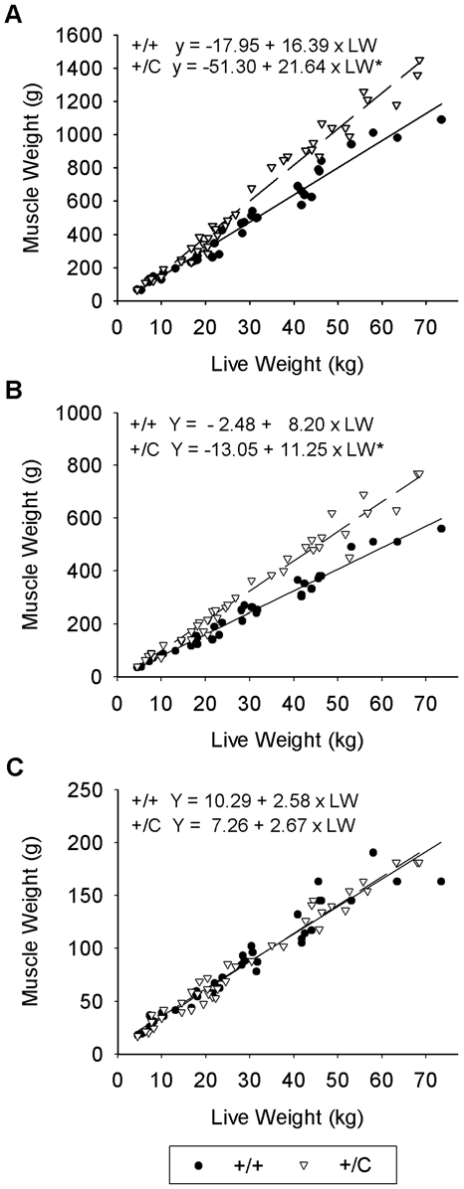
Muscle weight changes in callipyge (+/C) and normal (+/+) lambs. Muscle weights were regressed on live weight for the *longissimus dorsi* (A), *semimembranosus* (B) and *supraspinatus* (C). Equations are given for each muscle and genotype with an asterisk indicating statistical significance (P<.0001) in slopes and overall equations. There were no effects of genotype on the intercepts of any regression lines. The *longissimus dorsi* and *semimembranosus* muscles of callipyge lambs grew at a faster rate that normal lambs, but growth of the supraspinatus was not affected by genotype.

Phenotypic data for muscle weights of animals sampled for this experiment (Figure 2) show the characteristic muscle hypertrophy responses that have been previously reported for callipyge lambs [1]–[3]. The regression analysis of muscle weights on live weights show that callipyge (*+/C^Pat^*) *longissimus dorsi* (Fig. 2A) and *semimembranosus* (Fig. 2B) grew at a faster rate than normal (*+/+*) lambs after birth (P<0.0001 for both). The *supraspinatus* (Fig. 2C) regression shows no effect of genotype (P = 0.6999) at any live weight, making the *supraspinatus* an appropriate non-hypertrophied control muscle for the gene expression experiments. Furthermore, the intercept values for all three muscles were not significantly different between callipyge and normal animals indicating that the callipyge lambs have normal muscle development at birth and hypertrophy develops postnatally.

**Figure 3:**
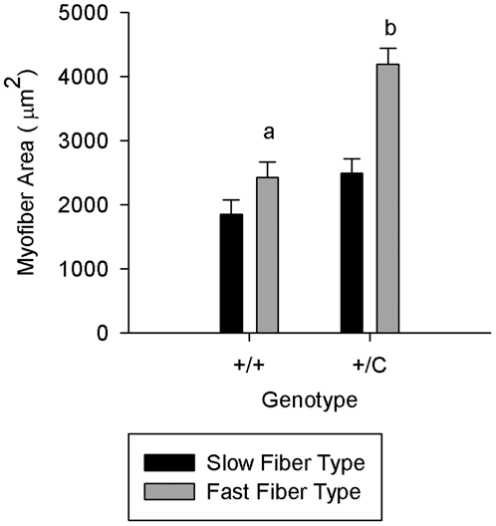
Myofiber area of *semimembranosus* muscles in callipyge and normal lambs. Cross sectional areas of fast and slow twitch myofibers of 20–30 day old callipyge and normal lambs are shown. Differing lower case letters indicate significance between genotypes (P = 0.0009). There was a trend (P = 0.0804) for the area of slow twitch fibers to be larger in callipyge lambs. These results indicated hypertrophic growth had begun in callipyge lambs at these ages.

One objective was to identify the early transcriptional changes that lead to muscle hypertrophy, so three of the four ages used for microarray analysis (10, 20 and 30 days of age) are prior to sufficient protein accretion (4–18 kg live weight) for muscle weights of callipyge lambs to be statistically heavier than normal lambs. Therefore, cross sectional areas of fast and slow twitch myofibers were measured in the *semimembranosus* muscle of 20 and 30 day old lambs as a more sensitive measure of the onset of muscle hypertrophy. Cross sectional areas and the proportion of fast and slow twitch myofibers were analyzed for the effect of age and genotype. There were no significant effects of age so the results shown (Fig. 3) were pooled from both ages. Callipyge (*+/C^Pat^*) lambs exhibited larger cross sectional areas of fast twitch fibers (P = 0.0009), which were on average 72% larger than normal (*+/+*) counterparts. The proportion of slow twitch fibers were not different (P = 0.2259) between genotypes but there was a trend for cross sectional area of slow twitch fibers (P = 0.0804) to be larger in callipyge lambs at these ages (Figure 3). The phenotypic data demonstrate that the process of hypertrophy had begun at these young ages and the complete sample collection is appropriate for microarray analysis and quantitative PCR validation of transcriptional changes that result in muscle hypertrophy.

### Paternal Callipyge Allele Study

This experiment compared gene expression in the callipyge (*+/C^Pat^*) and normal (+/+) genotypes over a developmental time course of four postnatal ages (10, 20, 30 and 80 D). Microarray analysis of the semimembranosus RNA (n = 16) on the bovine Affymetrix GeneChip produced signals from 16,369 probe sets that correspond to 16,034 Unigene transcripts. The Robust Multi-array Average (RMA) algorithm [49] identified 375 probe sets that were influenced by genotype *(+/C^Pat^* vs. *+/+*) and 111 probe sets with a significant effect of age at a False Discovery Rate (FDR)<0.10. Supplementary Table S1 provides the complete list of Affymetrix probe sets and gene identifications available from the NetAffx Analysis Center (Affymetrix Annotation Release 28 March 2009) There were no significant genotype by age interactions. Based on the transformed and normalized mean expression values for callipyge and normal genotypes, 212 probe sets (57%) had higher transcript abundance and 163 (43%) had lower transcript abundance in callipyge (*+/C^Pat^*) muscle relative to normal (+/+) muscle. The 375 Affymetrix probe sets produced 199 genes with functional annotations from the DAVID Knowledgebase using the gene conversion tool [50]. This data set was analyzed with the Functional Annotation Clustering tool in DAVID [51], [52] against the DAVID bovine background gene set which clustered between 113–139 genes into 48 functional groups (Supplementary Table S2). A summary of the gene ontology terms and their associated probe sets that were enriched by 2-fold or greater are shown in Table 1. This table is a summary of terms as the biological process and molecular function categories had many gene ontology terms that contained the same sets of genes with similar levels of enrichment. The summary shows enrichment of genes involved in respiration, glucose, lipid and energy metabolism as well as myosin contractile complexes, motor activity and kinase activity. Several functional clusters had distinct trends with respect to the callipyge genotype, revealing how several cellular systems are changing during muscle hypertrophy. All genes that have an electron transport function were decreased in callipyge muscle, as were the transcripts associated with the TCA cycle. Mitochondrial related transcripts and transcripts with oxidation-reduction activity were also decreased in callipyge muscle compared to wild-type. Functional groups of genes that were up-regulated in callipyge lambs included kinases, apoptotic factors, and glycolytic enzymes.

**Table 1:** Summary of functional annotation clusters for the effect of genotype.

Category	Total Genes^1^	GO Term	Description	Count	Fold Enrichment	Benjamini FDR	Probe Set ID
Cellular Component	113	016459	myosin complex	3	5.1	0.9801	Bt.1905.1.S1_at, Bt.4867.2.S1_at, Bt.12300.2.S1_at
		031980	mitochondrial lumen	8	3.1	0.5784	Bt.5966.1.A1_at, Bt.16082.1.S1_at, Bt.5108.1.S1_at, Bt.5161.1.S1_at, Bt.21849.1.S1_at, Bt.4555.1.S1_at, Bt.28162.1.S1_at, Bt.28162.2.A1_at, Bt.460.1.S1_s_at
Biological Process	121	045333	cellular respiration	4	5.2	0.9796	Bt.13324.2.S1_a_at, Bt.5520.1.S1_at, Bt.23357.1.S1_at, Bt.13128.1.S1_at,
		006094	gluconeogenesis	3	5.1	0.9999	Bt.562.1.S1_at, Bt.13505.1.S1_at, Bt.19161.1.S1_at,
		006006	glucose metabolic process	8	5.1	0.1762	Bt.23217.1.S1_at, Bt.25555.1.S1_at, Bt.3005.1.S1_at, Bt.562.1.S1_at, Bt.22169.1.S1_at, Bt.23399.1.S1_at, Bt.13505.1.S1_at, Bt.19161.1.S1_at
		032787	monocarboxylic acid metabolic process	13	4.3	0.0243	Bt.21113.1.S1_a_at, Bt.136.1.S1_at, Bt.562.1.S1_at, Bt.23607.1.S1_at, Bt.21584.1.S1_at, Bt.7215.1.S1_at, Bt.5520.1.S1_at, Bt.13505.1.S1_at, Bt.5966.1.A1_at, Bt.13324.2.S1_a_at, Bt.23094.4.S1_at, Bt.21849.1.S1_at, Bt.19161.1.S1_at
		009057	macromolecule catabolic process	9	2.6	0.9407	Bt.23217.1.S1_at, Bt.25555.1.S1_at, Bt.3005.1.S1_at, Bt.562.1.S1_at, Bt.22169.1.S1_at, Bt.23399.1.S1_at, Bt.13505.1.S1_at, Bt.15972.1.S1_at, Bt.5408.1.A1_at
		009790	embryonic development	4	2.3	1.0000	Bt.1151.1.S1_at, Bt.3730.1.S1_at, Bt.15972.1.S1_at, Bt.5226.1.S1_at
Molecular Function	139	003995	acyl-CoA dehydrogenase activity	3	8.4	0.9988	Bt.5966.1.A1_at, Bt.21849.1.S1_at, Bt.7215.1.S1_at
		016298	lipase activity	3	5.2	0.9997	Bt.5387.1.S1_at, Bt.448.1.S1_at, Bt.5223.1.S1_at
		050660	FAD binding	4	3.6	0.9993	Bt.5966.1.A1_at, Bt.21849.1.S1_at, Bt.7215.1.S1_at, Bt.4008.1.S1_at
		003774	motor activity	3	2.4	1.0000	Bt.1905.1.S1_at, Bt.4867.2.S1_at, Bt.12300.2.S1_at
		008234	cysteine-type peptidase activity	3	2.3	1.0000	Bt.13730.1.A1_at, Bt.13730.2.S1_at, Bt.393.1.S1_at, Bt.5408.1.A1_at
		004674	protein serine/threonine kinase activity	7	2.1	0.9997	Bt.17952.1.A1_at, Bt.120.1.S1_at, Bt.19447.1.A1_at, Bt.1.1.S1_at, Bt.4532.1.S1_at, Bt.4532.1.S2_at, Bt.7105.1.S1_at, Bt.8706.1.S1_at, Bt.22106.2.S1_at

1 Number of genes out of the 375 genes with a significant effect of genotype that have GO term annotation for each category.

**Figure 4:**
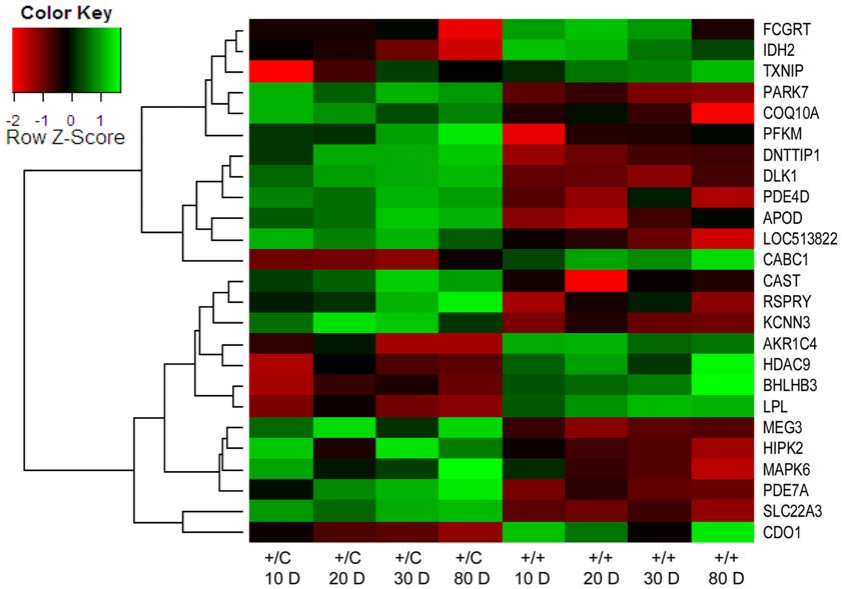
Hierarchal clustering and microarray signal intensity for genes validating an effect of genotype by quantitative PCR. Gene names are listed for rows and columns are fixed for age in days and genotype (+/C for callipyge and +/+ for normal). Relationship amongst genes sets was determined by hierarchal clustering in the heatmap.2 function of Bioconductor. Relative intensity by RMA is represented by green if expression was higher than average or red if expression was lower than average.

Quantitative PCR was used to verify differential gene expression between callipyge (*+/C^Pat^*) and normal lambs (*+/+*) on a larger sample collection that included the individuals used for the microarray analysis as well as additional lambs (biological replicates) and additional ages from prenatal to 150 days of age. Comparison of the current data set with published results indicated a number of previously known differentially expressed genes [47], [48] with FDR values greater than 0.05 (ATF4, COQ10A and HIPK2 with FDR = 0.0615, 0.1033 and 0.2177, respectively), therefore, we selected transcripts across a range of FDR values. The 49 probe sets that were tested by quantitative PCR are given in Table 2 along with their FDR values, gene symbols, descriptions and public database identifiers. Overall, 51% (25 of 49) of the transcripts had significant (P<.05) differential expression between genotypes by quantitative PCR. For probe sets with a FDR value of less than 0.01 or 0.05, differential gene expression between genotypes was verified for 70.5% (12 of 17) and 51.2% (20 of 39) of the transcripts, respectively (Table 2). Figure 4 shows the relative expression levels based on RMA interpretation of the 25 verified transcripts along with a hierarchical clustering of these transcripts. Sixteen of the transcripts had higher abundance in callipyge (*+/C^Pat^*) muscle and 9 transcripts had higher abundance in normal (+/+) muscle. Transcripts that were verified to be differentially expressed in *semimembranosus* were subsequently tested in *longissimus dorsi* and *supraspinatus* muscle samples. No further quantitative PCR analysis was conducted if differential expression of transcripts identified in this study were not verified in the *semimembranosus* RNA. The complete summary of quantitative PCR statistics for the main effects of genotype and age as well as the genotype by age interaction in all three muscles are given in Supplementary Table S3. Quantitative PCR data for transcript abundance (least square means and standard errors) in the *semimembranosus* and *supraspinatus* can be found in Supplementary Tables S4 and S5, respectively.

**Table 2:** Affymetrix probe sets that were validated by quantitative PCR.

Probe Set ID	Genotype FDR^1^	Entrez Gene ID^2^	Gene Symbol	Gene Description
Bt.23094.4.S1_at	0.0518	282138 782922	AKR1C4 LOC782922	aldo-keto reductase family 1, member C4
Bt.13975.1.S1_a_at	0.0377	613972	APOD	apolipoprotein D
Bt.12583.1.A1_at	0.0329	613907	BHLHB3	basicHLH Class B3, DEC2, SHARP-1
Bt.22379.1.S1_at	0.0099	536925	CABC1	chaperone, ABC1 activity of bc1 complex homolog (S. pombe)
Bt.4738.1.S1_at	0.0076	281039	CAST	calpastatin
Bt.2723.1.A1_at	0.0661	514462	CDO1	cysteine dioxygenase, type I
Bt.5223.1.S1_at	0.0014	281117	DLK1	delta-like 1 homolog (Drosophila)
Bt.8972.1.S1_at	0.0014	505524	DNTTIP1	deoxynucleotidyltransferase, terminal, interacting protein 1
Bt.1035.1.S1_a_at	0.0105	338062	FCGRT	Fc fragment of IgG, receptor, transporter, alpha
Bt.17876.1.A1_at	0.0478	CK847416	HDAC9	similar to histone deacetylase 9
Bt.5520.1.S1_at	0.0218	327669	IDH2	isocitrate dehydrogenase 2 (NADP+), mitochondrial
Bt.26158.1.A1_at	0.0010	534180	KCNN3	potassium intermediate/small conductance calcium-activated channel, family N3
Bt.26750.1.S1_at Bt.26750.2.A1_at	0.0099 0.0518	513822	LOC513822	hypothetical protein containing a methyltransferase domain
Bt.5387.1.S1_at	0.0046	280843	LPL	lipoprotein lipase
Bt.3448.3.S1_at	0.0288	538094	MAPK6	mitogen-activated protein kinase 6
Bt.12510.1.S1_at	0.0038	CB464112	MEG3	maternal expressed gene 3, GTL2 variant D
Bt.21745.1.S1_at	0.0010	511268	PARK7	Parkinson disease (autosomal recessive, early onset) 7
Bt.20446.1.A1_at	0.0010	539556	PDE4D	cAMP specific phosphodiesterase 4D
Bt.12327.1.S1_at	0.0046	506790	TXNIP	thioredoxin interacting protein
Bt.14371.1.A1_at	0.0099	CK968244	SLC22A3	similar to solute carrier family 22 member 3
Bt.2347.3.S1_a_at	0.0187	506544	PFKM	phosphofructokinase, muscle
Bt.19420.1.S1_at	0.0343	CB464927	PDE7A	similar to phosphodiesterase 7A
Bt.9244.1.A1_at	0.0518	538571	RSPRY1	ring finger and SPRY domain containing 1
Bt.27381.1.S1_at	0.1033	538821	COQ10A	coenzyme Q10 homolog A (S. cerevisiae)
Bt.6890.1.S1_at	0.2177	CB439427	HIPK2	homeodomain interacting protein kinase 2
Bt.12510.1.S1_at^3^	0.0016^4^	CB464112	MEG3	maternal expressed gene 3/gene trap locus 2 variant D
Bt.18078.1.S1_at^3^	0.0829^4^	CB439344	MEG8	maternal expressed gene 8

1 Genotype FDR from the microarray analysis. Statistics for all genes tested by quantitative PCR are given in Supplementary Table S1 (paternal allele study) and Supplementary Table S4 (maternal allele study).

2 Most representative public sequence identification is given if no Entrez gene ID was available.

3 Probe sets that were validated from the maternal allele genotype comparisons.

4 FDR value shown was for *C^Mat^/+* - +/+ comparison.

**Figure 5:**
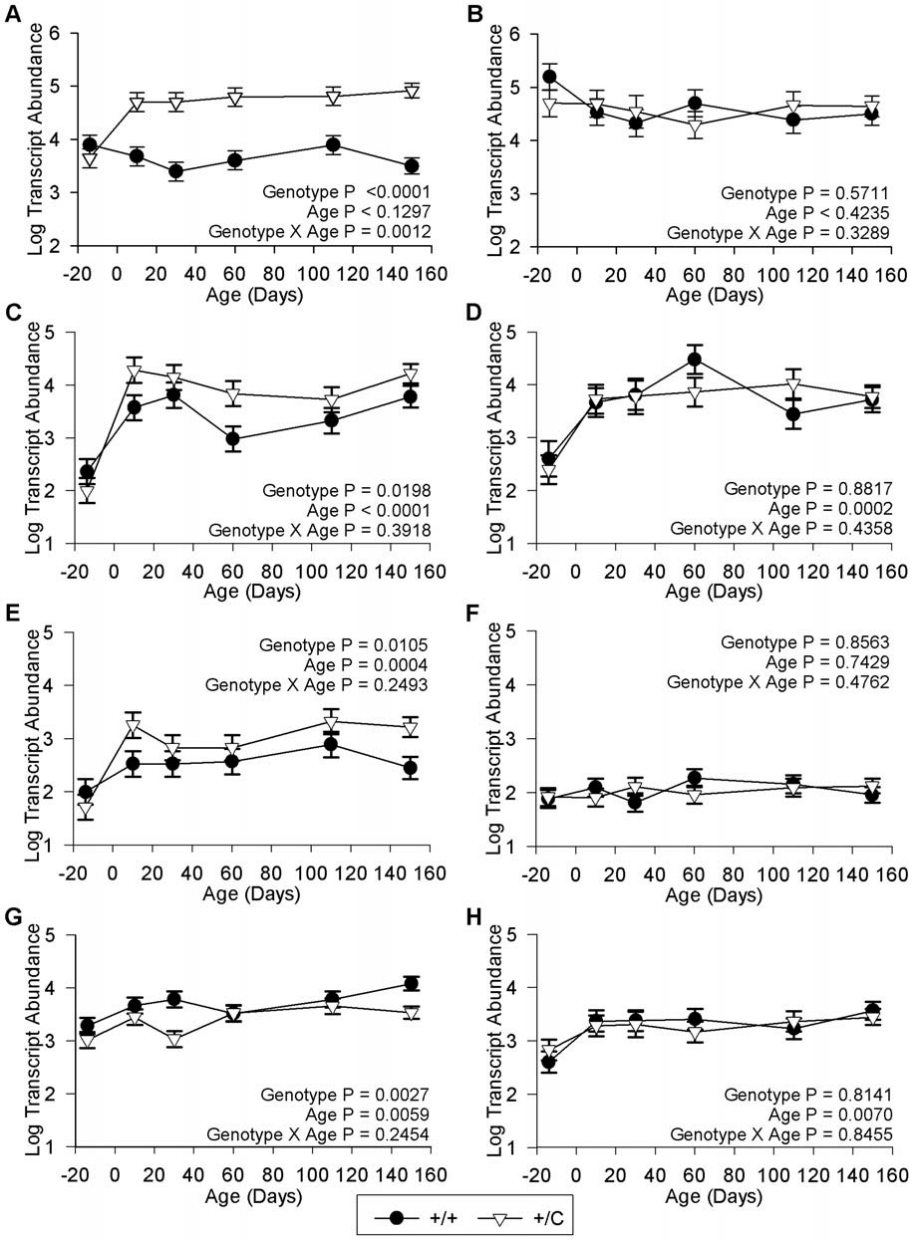
Candiate genes with an expression pattern resembling *DLK1*. Least square means and standard errors for log transcript abundance in 100 ng of total RNA in a hypertrophied muscle (*semimembranosus*, A, C, E, G) and a non-hypertrophied muscle (*supraspinatus*, B, D, F, H) in callipyge (+/C) and wild-type (+/+) lambs. Birth is day 0 and samples from 14 days prepartum are included and represented as -14 days. *DLK1* expression is shown in A and B. Transcripts *PDE4D* (C and D), *PARK7* (E and F) and *BHLHB3* (G and H) show a significant effect of the callipyge genotype in *semimembranosus*, while no effect is observed in the *supraspinatus*. This pattern of expression suggests these genes are responding to DLK1 signaling in callipyge muscle.

Several studies have demonstrated that *DLK1* and *RTL1* are highly up-regulated in the hypertrophied muscles of callipyge (*+/C^Pat^*) sheep [18]–[20], [47]. The microarray analyses (Fig. 4) and quantitative PCR results confirmed a significantly higher transcript abundance of *DLK1* (P<.0001) in the *semimembranosus* (Fig. 5A) and *longissimus dorsi* (Supplementary Table S1) of callipyge (*+/C^Pat^*) lambs relative to normal (+/+) lambs. There was no effect of genotype (P = .5711) on the expression of *DLK1* in the *supraspinatus* (Fig. 5B). Figure 5 shows developmental gene expression data for three genes that have similar expression profiles to *DLK1*. Two of these transcripts, *PDE4D* (Fig. 5C and D) and *PARK7* (Fig. 5E and F), had significantly increased transcript abundance in the *semimembranosus* and *longissimus dorsi* of callipyge (*+/C^Pat^*) lambs but no significant differences in the *supraspinatus*. Ten other verified genes had a similar expression pattern including *COQ10A*, *DNTTIP1*, *HDAC9*, *HIPK2*, *LOC513822*, *MAPK6*, *MEG3*, *PFKM*, *RSPRY1* and *SLC22A3* (Table 2, Supplementary Table S3). Four verified transcripts were significantly down-regulated in the *semimembranosus* and *longissimus dorsi* of callipyge (*+/C^Pat^*) lambs relative to normal (+/+) lambs but no effect of genotype was detected in the *supraspinatus*. These genes included *BHLHB3* (Fig. 5G and H), *AKR1C4*, *CDO1*, *IDH2* and *LPL* (Table 2, Supplementary Table S3).

**Figure 6:**
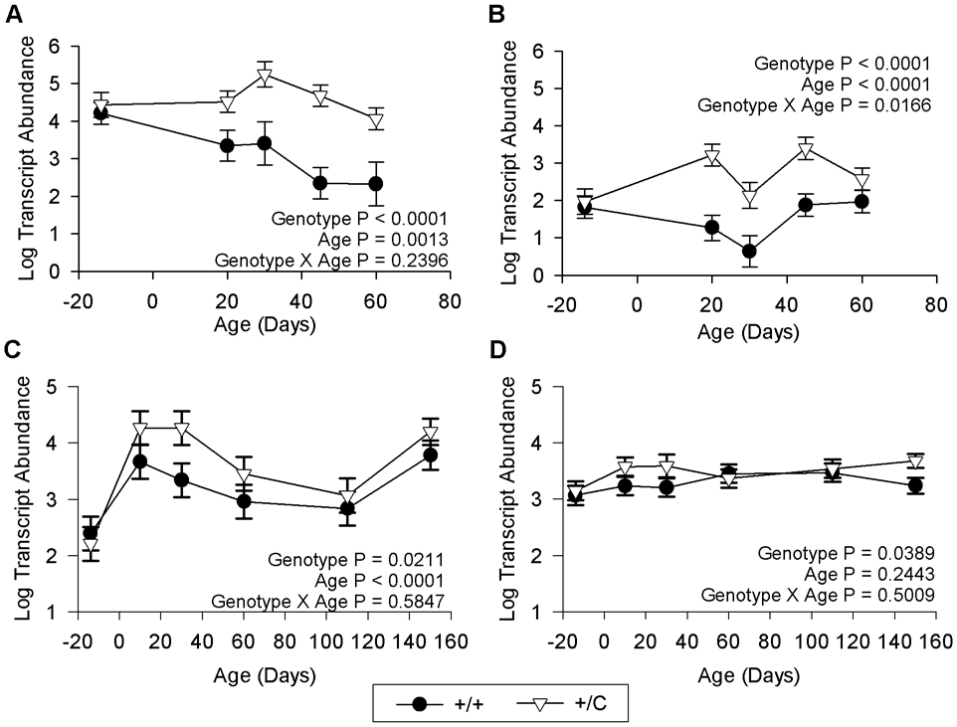
*PDE7A* exhibits an expression pattern resembling *RTL1*. Least square means and standard errors for log transcript abundance in 100 ng of total RNA in a hypertrophied muscle (*semimembranosus*, A and C) and an unaffected muscle (*supraspinatus*, B and D) in callipyge (*+/C*) and wild-type (+/+) lambs. Birth is day 0 and samples from 14 days prepartum are included and represented as -14 days. *RTL1* expression is shown in A and B and *PDE7A* expression is shown in C and D. Transcript abundance of *RTL1* and *PDE7A* is up-regulated as a result of the callipyge allele in both muscles, suggesting *PDE7A* may respond to *RTL1* expression.

Probe sets for *RTL1* were not represented on the bovine Affymetrix GeneChip but quantitative PCR analysis showed significant up-regulation of *RTL1* (P<.0001) in the *semimembranosus* and *supraspinatus* (Fig. 6A and B) consistent with previous results [18], [20]. Elevated levels of *RTL1* expression have been demonstrated in the *longissimus dorsi* of callipyge (*+/C^Pat^*) lambs [18] but was not verified for this study. One transcript, *PDE7A*, was significantly up-regulated in the *semimembranosus*, *longissimus dorsi* (P<.0001 for both), and the *supraspinatus* (P = 0.0389) of callipyge (*+/C^Pat^*) animals (Fig. 6C and D) suggesting that *PDE7A* may be a down-stream target of *RTL1* in skeletal muscle. Two additional genes, *APOD* and *KCNN3* were also significantly more abundant in *semimembranosus* and *longissimus dorsi* but had a trend for differential expression (P = 0.0718 and 0.0994, respectively) in the *supraspinatus* (Supplementary Table S3).

Five other transcripts that were validated by quantitative PCR were only differentially expressed in one of the hypertrophied muscles indicating that they are indirect and muscle-specific responses to *DLK1* or *RTL1*. The transcript abundance of *FCGRT* and *TXNIP* were significantly down regulated (P = 0.0228 and 0.0002, respectively) in the *semimembranosus* of callipyge (*+/C^Pat^*) lambs but was not differentially expressed in the *longissimus dorsi* or *supraspinatus* (Supplementary Table S3). Transcripts for *ATF4* and *RPS6K* have been shown to be differentially expressed in the *longissimus dorsi* [47], [48] which was verified in this study but the two transcripts showed no effect of genotype in the *semimembranosus* or *supraspinatus* (Supplementary Table S3).

### Maternal Callipyge Allele Influence

**Figure 7:**
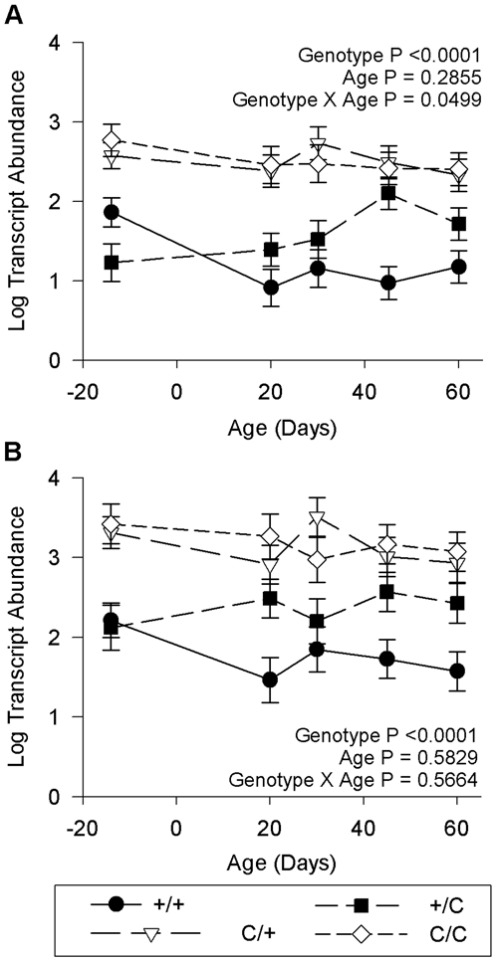
Expression of *MEG8* and CB439344 in the *semimembranosus* influenced by the presence of a maternal callipyge allele. Expression of *MEG8* exons in all four possible callipyge genotypes (A) is shown in comparison to expression of transcript CB439344 (B) that maps to the C/D snoRNA clusters of *MEG8* in cattle. Lambs possessing a maternal callipyge allele (*C/+* and *C/C*) exhibit significantly higher levels of *MEG8* expression and CB439344 than callipyge (*+/C*) and normal lambs (*+/+*).

In the microarray experiment contrasting all four possible genotypes, nine transcripts were detected as differentially expressed in animals possessing a maternal callipyge allele (*C^Mat^/+* and/or *C/C)*. However upon quantitative PCR validation of all nine transcripts, only three exhibited a significant change in expression over several ages (Supplementary Tables S6 and S7). The *PARK7* transcript was differentially expressed in the *C/C* -*C^Pat^*/+ contrast, however this transcript was also differentially expressed in the paternal allele analysis (*+/C^Pat^* vs +/+) so this was interpreted to be an effect of the paternal callipyge allele in the homozygous (*C/C*) animal. The other two transcripts, *MEG3* and CB439344, were both located within the *DLK1-DIO3* domain. The CB439344 transcript lies within a cluster of C/D snoRNA on bovine chromosome 21 (Figure 1; maternal allele; black diamond) which are found in the *MEG8* gene of humans [38]. The expression of CB439344 is nearly identical in magnitude and pattern of response to the callipyge mutation compared to *MEG8* as measured by quantitative PCR (Fig. 7). Muscle samples from lambs of both genotypes possessing a maternal copy of the callipyge allele (*C^Mat^/+* and *C/*C) express significantly higher levels of both *MEG8* and CB439344 (P<0.0001; Figure 7; Supplementary Table S6). The *MEG8* and CB439344 transcripts were also differentially expressed in the supraspinatus (Supplementary Table S6) which has been previously demonstrated for *MEG8* [20]. This shows that CB439344 is directly influenced in *cis* by the callipyge SNP that is 200 kb away and is consistent with the proposal that *MEG8* transcribes a very long non-coding RNA which includes the C/D snoRNA in sheep.

One transcript, similar to selenophosphate synthetase 2 *(SEPHS2)*, was up-regulated in the *C/C* semimembranosus, but only at 30 days of age. This result was confirmed by qPCR analysis. (data not shown). Thus, the overall effect of genotype was non-significant across all ages as measured by qPCR. No other significant effect of the maternal callipyge allele was detected (Supplementary Table S6).

## Discussion

Callipyge sheep are a unique model for investigating the effects of elevated expression of the paternal and maternal allele-specific transcripts from the *DLK1-DIO3* domain. All of the lambs in this study were actively growing due to the normal neuronal, hormonal, nutritional, and exercise stimuli present in young animals. Callipyge lambs have normal muscle development at birth and just prior to birth, no differences in *DLK1* and *RTL1* expression were detected [20]. One possibility is that mechanical tension and nerve stimulation that occurs when lambs begin to walk and run is the initiating stimulus for differential *DLK1* and *RTL1* expression and the onset of muscle hypertrophy.

Changes in gene expression during muscle hypertrophy can be categorized as the primary causative genes, *DLK1* and *RTL1*, which are known due to the inheritance model, the secondary effector genes that have a direct transcriptional response to *DLK1* and *RTL1* activities and the tertiary responses associated with hypertrophy such as protein accretion, myofiber type and metabolic changes. The mechanisms by which elevated *DLK1* and *RTL1* protein levels initially induce changes in gene expression are not likely to be detected by RNA analysis but the changes in transcript abundance of the secondary and tertiary genes can be collectively identified by gene expression profiling. This study of the *semimembranosus* muscle, along with our previous analysis in the *longissimus dorsi* muscle [47], focused the majority of the microarray analysis on early muscle hypertrophy (10, 20, and 30 days) in an effort to be sure to identify the secondary effector genes that respond to *DLK1* and/or *RTL1* expression. Our initial analysis of the onset of muscle hypertrophy in the *longissimus dorsi* (10, 20 and 30 days) identified 13 probe sets with an effect of genotype [47]. Our results in the *longissimus dorsi* and *semimembranosus* (current study) indicate that changes in gene expression and phenotype have begun at these ages. One older time point, 80 days of age, was included for well-established hypertrophy in the current study and the broadest inclusion of the tertiary responses. Inclusion of this older age as well as the use of different image interpretation algorithms (MAS5 versus RMA) may account for the large increase to 375 probe sets with a significant effect of genotype.

A quantitative PCR validation strategy using a larger data set and three muscles was used to help define secondary and tertiary responses in hypertrophy. We are making the assumption that changes in transcript abundance will correspond to altered protein expression and biological activity, however, we recognize that will not be true for all genes.

Comparisons of the microarray results from Fleming-Waddell et al, [47]and the current results show an overlap of 8 transcripts affected by genotype (*DLK1*, *DNTTIP1*, *LOC513822*, *COQ10A*, *PDE7A*, *PFKM*, *RSPRY1*, and *RPS6KA3*) in both muscles. Seven of the transcripts were verified to be differentially expressed in the *longissimus dorsi* and *semimembranosus* by quantitative PCR with the exception being *RPS6KA3*, where differential expression was only verified in the *longissimus dorsi*. Another analysis of gene expression in the *longissimus dorsi* of callipyge sheep examined neonatal animals (less than 5 days of age) and 80 days when muscle hypertrophy has been well established [48]. This study used technical replicates of pooled RNA from different animals and analyzed differences between genotypes at each age as well as contrasts of normal muscles with different fiber type compositions. All three studies produce different but overlapping groups of genes that are influenced by the callipyge genotype and provide new insights into the potential mechanism of *DLK1* and *RTL1* to alter skeletal muscle growth.

The predominant changes in gene expression identified by cluster analysis were metabolic biological processes as well as changes in the mitochondria and myosin complex. This result was greatly influenced by the state of annotation of the transcripts for the cattle as only 199 of 375 probe sets had sufficient annotation for cluster analysis by DAVID. Included in these metabolic genes are two key rate-limiting allosteric enzymes. The enzyme isocitrate dehydrogenase 2 (*IDH2*) catalyzes the first oxidation-reduction step in the citric acid cycle. This is a key component in the oxidative phosphorylation pathway and is significantly decreased in callipyge hypertrophied muscles. In contrast, muscle phosphofructokinase (*PFKM*) is the first committed rate limiting step for glycolysis. *PFKM* expression is highly up-regulated in callipyge skeletal muscle. The expression profile of these two enzymes confirms a shift from oxidative phosphorylation to glycolysis in the muscle as a result of increasing the proportion of type IIB glycolytic myofibers in callipyge sheep [53], [54]. The differences observed in these metabolic transcripts are likely tertiary effects of the fiber type change.

Several candidate genes in our list of validated transcripts suggest that callipyge muscle hypertrophy may be the result of a loss of repression or reduction in a negative feedback mechanism for controlling normal growth signals. Our data suggest that the secondary effector genes may be nuclear regulatory factors (chromatin modifiers, transcription factors or transcription accessory factors) and components of signal transduction pathways.

Two known transcriptional repressors of myogenesis, *HDAC9* and *BHLHB3* (also known as *SHARP1*; *DEC2*) were down-regulated in callipyge muscle. The role of *HDAC9* in the feedback inhibition of myogenic differentiation has been described [55] and a model for the mechanism of *DLK1* inhibition of *HDAC9* in callipyge muscle has been proposed [48]. *BHLHB3* is a transcriptional repressor of genes containing E-box elements [56]-[60] and along with *BHLHB2* (also known as *SHARP2*; *DEC1*) are members of a fifth circadian clock gene family [61], [62]. *BHLHB3* is in the same family of class B3 proteins as *HES-1*, a downstream target of Notch-1 activation which also inhibits the myogenic regulatory factor, MyoD [63]–[65]. Specifically in muscle, *BHLHB3* exhibits negative regulation of MyoD activation of myogenin transcription in skeletal muscle [66]. Lower levels of *BHLHB3* in callipyge muscle would promote the rate of fusion of individual myoblasts into mature myofibers [67]. DLK1 is thought to act as a ligand for Notch-1 [68] and has been shown to antagonize Notch-1 activity [68]–[70]. Down-regulation of BHLHB3 may be an early indicator of suppression of Notch-1 signaling in callipyge muscle. The chromosomal region on bovine chromosome 5 that contains *BHLHB3* also contains several significant quantitative trait loci (QTL) for muscle growth including dressing percentage and *longissimus dorsi* rib eye area [71].

Several transcripts that were confirmed as differentially expressed between callipyge (*+/C^Pat^*) and normal (+/+) muscles have regulatory roles in the β-adrenergic or mTOR/AKT signaling pathways that control postnatal muscle growth. Two phosphodiesterase transcripts, *PDE7A* and *PDE4D*, were validated by quantitative PCR and a third, *PDE8A* was identified by microarray analysis but not validated. All three are cAMP-specific phosphodiesterases that hydrolyze cAMP into the inactive substrate 5′-AMP and therefore have a role in regulating the duration and magnitude of cAMP second messenger activities [72]–[74]. Both *PDE7A* and *PDE4D* are members of multi-gene families and each produces a series of splice variants that determine their subcellular location and compartmentalize their effects on cAMP signaling. Identifying the specific variants that are up-regulated and their localization in muscle will be essential to determine their role in muscle hypertrophy.

*PDE4D* accounts for 80% of the phosphodiesterase activity in skeletal muscle and plays a role in lipolysis rates [75]. Expression of *PDE4D* was required for IGF-1 induced myogenic differentiation in rat L6 myoblast cells [76]. *PDE4D* is a component of the β_2_-adrenergic receptor signaling complex in cardiac muscle [77] and has a role preventing the desensitization and down regulation of the β_2_-adrenergic receptor [78], [79]. The up-regulation of *PDE4D* in callipyge muscle could prevent desensitization of the β_2_-adrenergic receptor that would propagate a stronger response to physiological levels of adrenaline in young growing lambs. The phenotype observed in callipyge hypertrophy is very similar to muscle growth induced by β-agonists, which includes an increase in size and proportion of fast twitch myofibers and a decrease in body fat content [80]-[82]. Callipyge sheep do not respond to β-agonist supplementation with additional muscle hypertrophy [80].

*PDE7A* splice variants are expressed in many tissues and are most abundant in skeletal muscle. Messenger RNA levels of *PDE7A* are much higher than corresponding protein levels in the same tissue, suggesting *PDE7A* is regulated at the post-transcriptional and translational level [83]. The quantitative PCR validation showed that *PDE7A* transcripts were up-regulated in all three muscles including the *supraspinatus* which has only has elevated levels of *RTL1* and does not become hypertrophied. This makes *PDE7A* a candidate to be a secondary effector gene since it responds to *RTL1* expression but is not confounded by muscle hypertrophy. Two other transcripts, *APOD* and *KCNN3*, showed a genotypic trend (P<0.10) for differential expression in the *supraspinatus* as well. The up-regulation of *PDE7A* in the supraspinatus is an indicator that *RTL1* expression has a biological activity in skeletal muscle and provides further evidence of its role in the callipyge phenotype beyond the *trans*-interaction of *RTL1* and *RTL1AS* microRNA as a mechanism of polar overdominance [24].

**Figure 8:**
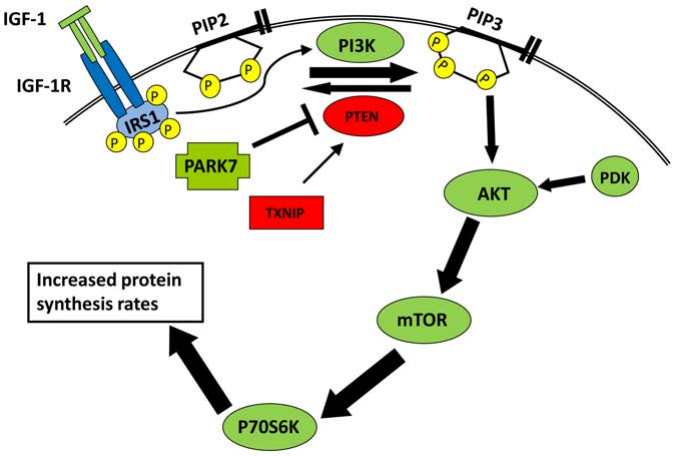
Influence of PARK7 and TXNIP on AKT/mTOR signaling in callipyge muscle hypertrophy. The diagram depicts the AKT response to IGF-1 signaling which increases protein synthesis. Two regulatory proteins of the AKT/mTOR pathway are differentially expressed in callipyge hypertrophied muscles. Factors that are inhibitory of protein synthesis are indicated in red and stimulatory factors are green. Transcripts that were down-regulated in callipyge (+/C^Pat^) muscle are rectangles and transcripts that were up-regulated in callipyge muscle are cross shaped. In this model, the phosphatase activity of PTEN is inhibited by PARK7 and TXNIP and the AKT/mTOR response to normal physiological levels of growth factors is magnified to increase protein synthesis rates.

The AKT/mTOR pathway has been shown to be an important signal transduction pathway for regulating muscle growth [63], [84]–[88]. Two transcripts were validated by quantitative PCR, *PARK7* and *TXNIP*, are components of a feedback mechanism controlling AKT activation. Figure 8 shows a working hypothesis for how this pathway may be affected in callipyge lambs. A variety of hormone -receptor interactions, β-adrenergic, insulin or IGF-1 (IGF-1 depicted in Figure 8) stimulate the phosphoinositide-3-kinase (PI3K) pathway to produce phosphatidylinositol (3,4,5) triphosphate (PIP_3_). PTEN (phosphatase and tensin homologue) is a 3′ phosphatase that regulates the pool of PIP_3_ by catalyzing dephosphorylation of PIP_3_ to phosphatidylinositol (3,4) diphosphate (PIP_2_). AKT (also known as protein kinase B) is recruited to the membrane by PIP_3_ and activated via phosphorylation by phosphoinositide-dependent kinase (PDK). Activated AKT can phosphorylate a number of target proteins including mTOR (mammalian target of rapamycin) which in turn activates p70^S6K^ to increase protein synthesis [89].

The PARK7 protein, also known as DJ-1, has been shown to negatively regulate the function of PTEN [90]. PARK7 was up-regulated in both the *semimembranosus* and *longissimus dorsi* which would result in less PTEN phosphatase activity and a higher level or longer duration of AKT activation after growth factor stimulation. Mouse models of constitutively active AKT induce hypertrophy, specifically in the fast twitch Type IIB fibers of skeletal muscles and cause a reduction in body fat similar to the callipyge phenotype [87], [88]

*TXNIP* was significantly decreased in callipyge *semimembranosus* and is a negative regulator of protein synthesis. The TXNIP protein reduces thioredoxin in the cell [91] which is necessary for the reactivation of oxidized PTEN [92], [93]. Therefore reduced levels of TXNIP would decrease the activity of PTEN, allowing an increase in the amount of PIP_3_ available to activate signaling in the AKT/mTOR growth pathway [94]. *TXNIP* knock-out mice show high levels of accumulated inactive PTEN with higher levels of activated AKT resulting in more glycolytic oxidation in their skeletal muscle [94]. Low endogenous levels of *TXNIP* expression caused by genetic variation in pigs were correlated with heavier carcass weights and faster rates of gain in commercial swine breeds [95]. It should be noted that *TXNIP* expression levels were only significantly decreased in callipyge *semimembranosus*, and not in the *longissimus dorsi* indicating that this gene is a muscle specific tertiary response. The altered expression of both *PARK7* and *TXNIP* may explain why semimembranosus muscle undergoes the greatest magnitude of hypertrophy of all muscles in callipyge animals [2], [4], [5].

Gene expression in the two genotypes that have a maternal callipyge allele (*C^Mat^/+* and *C/C*) were compared to both callipyge (*+/C^Pat^*) and normal (+/+) *semimembranosus* to examine the effects of over-expression of the maternal transcripts and their small RNA. Microarray analysis identified differential expression of 9 transcripts at a single age. However, only transcripts located within the *DLK1-DIO3* domain were verified by quantitative PCR to have a significant effect of the maternal callipyge allele in animals from 14 days prenatal to 60 days of age. Notably, the transcript annotated as CB439344 occurs nearly 90 kb downstream of the reported end of the four *MEG8* exons in sheep and cow. This transcript falls within a region of C/D snoRNA that were identified in the human *MEG8* [38] and are present in the bovine genome sequence. Our data extend the *cis* effect of the callipyge mutation into the C/D snoRNA clusters hosted in *MEG8* introns and support the hypothesis of a long maternal RNA transcript [39] through this region that could also include the microRNA cluster. The sequence of these C/D snoRNA in humans were not similar to other C/D snoRNA that are methylation guides for rRNA or U snRNA and may have a function other than RNA modification [38]. Target prediction of the microRNA from this domain in mouse and human indicates that they would target a broad variety of genes and not target a specific set of genes [42], [44]. We find no evidence that any genes expressed in *semimembranosus* and detectable by the Affymetrix bovine microarray are affected by these maternal allele specific transcripts. This does not rule out the possibility that these genes may have roles in early embryonic development or in the brain [39], [42].

This work has identified several hundred transcripts that were differentially expressed in the callipyge model and 25 genes have been validated by quantitative PCR in a larger sample population. Within this subset, we have identified changes in nuclear repressor expression and feedback mechanisms in two major protein accretion pathways, the β-adrenergic and AKT/mTOR, that were altered in callipyge hypertrophied muscles. The callipyge phenotype in sheep indicates that a significant potential for increased muscle growth remains in livestock without increasing feed intake or body size. The genes identified in this model are primary candidates for naturally regulating postnatal muscle growth in all meat animal species, and may serve as targets to ameliorate muscle atrophy conditions including myopathic diseases and age-related sarcopenia.

## Materials and Methods

### Ethics Statement

All animals were euthanized in a humane manner in accordance with approved Purdue University or Utah State University Animal Care and Use Committee protocols.

### Sample Collection

Planned matings were conducted to produce all four possible genotypes with respect to the callipyge allele. All lambs were genotyped at the callipyge single nucleotide polymorphism (Freking *et al.* 2002; Smit *et al.* 2003) and several microsatellite markers flanking the callipyge locus on chromosome 18. Callipyge (*+/C^Pat^*) and normal (*+/+)* lambs were sampled at 14 days prenatal and 10, 20, 30, 45, 60, 80, 90,110, 130, and 150 days post-partum with at least 3 animals per age and genotype combination for a total of 45 callipyge and 43 normal sheep. At 70 days of age, lambs were placed in individual pens to measure weekly weight gain and *ad libitum* feed consumption. Maternal heterozygotes (*C^Mat^/+*; n = 24) and homozygous (*C/C*; n = 19) lambs were sampled at 14 days prenatal, and 20, 30, 45, and 60 days of age. Lambs were weaned at 60 days of age and placed on a complete mixed ration fed *ad libitum*.

*Longissimus dorsi*, *semimembranosus*, and *supraspinatus* were excised and weighed. *Semimembranosus* samples were frozen in isopentane chilled in liquid nitrogen and sectioned in a cryostat microtome for immunofluorescence detection of fast and slow myofibers. The sections were fixed, stained and measured for cross sectional area using the methods previously described by White et al, [8].

Muscle samples were fixed in RNAlater storage solution (Ambion Inc., Woodlands, TX, USA) immediately after collection. Tissues were homogenized in 4 M guanidinium thiocyanate, 25 mM sodium citrate, 50 mM EDTA, 1% sodium-N-lauroyl-sarcosine. RNA was isolated by ultracentrifugation of the tissue homogenate on a cushion of 5.7 M CsCl, 50 mM EDTA, [96] and then further purified and treated with DNase I using NucleoSpin RNA II columns (Machery-Nagel Inc., Easton, PA, USA).

### Microarrays

RNA samples isolated from the *semimembranosus* muscles of two callipyge and normal lambs at 10, 20, 30 and 80 days of age were used for the paternal callipyge allele microarray experiment for a total of 16 individuals. For the maternal callipyge allele experiment, the *semimembranosus* of three maternal heterozygous (*C^Mat^/+*) and three homozygous (*C/C*) lambs at 30 days of age were used to compare to the 30 day old callipyge (*+/C^Pat^*) and normal microarrays already obtained. Each RNA sample was hybridized to a separate array, resulting in biological replicates for each genotype and age combination. Five micrograms of skeletal muscle total RNA were converted to biotinylated cRNA using the manufacturer's reagents and protocols (Affymetrix Inc., Santa Clara, CA, USA). GeneChip poly-A control factors were included in the cRNA synthesis and 20 µg of cRNA were fragmented and hybridized to the Affymetrix Bovine Expression Array. Eukaryotic hybridization controls and herring sperm DNA were added to the hybridization mix and incubated for 16 h at 45°C. Chips were then stained using streptavidin phycoerytherin. The arrays were scanned using the GeneChip Scanner 3000 and GeneChip Operating Software (GCOS, Affymetrix Inc.). All raw microarray data were deposited in Gene Expression Omnibus (NCBI, Bethesda, MD, USA) in accordance with MIAME compliance (accession no. GSE11780).

### Microarray Analysis

Affymetrix microarray images were interpreted by RMA [49], using Bioconductor in R [97], [98]. RMA values were directly placed into the second stage of the model proposed by Wolfinger [99] since RMA incorporates a transformation and normalization step. The second stage fit a separate model for each gene, allowing each gene its own variability estimate. The ANOVA performed in this step was a mixed model with genotype, age and genotype×age interaction as fixed effects and chip (nested within interaction) as a random effect. Using the p-values obtained from the second stage model for each gene, the Benjamini and Hochberg false discovery rate method [100] was employed to identify genes with significant differential expression between genotype, age, and their interaction. All microarray data analyses were performed in SAS (SAS Institute Inc., Cary, NC, USA).

The microarray analysis for the four genotype comparison used the same steps for image interpretation and the first stage normalization. An ANOVA was performed for the effect of genotype with the following specific pair-wise contrasts: *C/C* vs. *+/C^Pat^*, *C/C* vs. *C^Mat^/+*, and *C^Mat^/+* vs. *+/+*. Each contrast was subjected to its own FDR correction, as described previously.

### Quantitative RT-PCR

Primary annotations of probe set sequences were obtained from the Affymetrix NetAffx Analysis Center. Probe set sequences were also compared to the nonredundant Genbank database by BLAST (NCBI, Bethesda, MD, USA) to confirm the current most representative public ID and annotation for cattle. Transcripts will be discussed by gene name if known, or by bovine locus designation. All annotations and primer designs were performed using bovine sequences. Primers were designed for quantitative PCR (qPCR) from the most representative public ID sequence using Oligo5.0 software (Molecular Biology Insights, Inc., Cascade, CO). All quantitative PCR assays were developed with at least one primer contained within the probe set identity region of the bovine transcript. Primer sequences and quantitative PCR conditions are given in Supplemantary Table 8.

Complimentary DNA (cDNA) was synthesized and diluted according the methods described previously [47]. Primer pairs for qPCR analysis were tested on muscle cDNA from eight individuals using iQ SYBR Green Supermix reagents on an iCycler Real-Time PCR Detection System (Bio-Rad Inc., Hercules, CA, USA). Primer specificity and capture temperature were determined by melt curve analysis. PCR products were cloned into pCR-4TOPO vector and chemically transformed into TOP10 *E. coli* (Invitrogen, Inc). Plasmids were sequenced to confirm that the sheep ortholog of each respective bovine transcript was amplified by the primers. Plasmids with sequence-verified inserts were quantified by fluorometry (Picogreen® dsDNA Quantitation Kit, Invitrogen, Inc) and digested with *Eco*RI.

Quantitative PCR assays were carried out in 15 µL reaction volumes of iQ SYBR Green Supermix with diluted first-strand cDNA equivalent to 100 ng of input RNA. All cDNA samples were assayed in duplicate. Primer sequences and thermal cycling conditions are given in Supplemantary Table 8. Quantification standards were comprised of four 100-fold dilutions of plasmid DNA (10^8^ to 10^2^ or 10^7^ to 10^1^ molecules) and were assayed in triplicate. Standards were used to calculate a linear regression model for threshold cycle relative to transcript abundance in each sample.

Log values for transcript abundance from each sample duplicate were subjected to an ANOVA using the MIXED procedure of SAS® for genotype and age effects as well as the genotype×age interaction. The random effect was defined as animal nested within genotype×age. An alpha of 0.05 was used for all PCR analysis and any significant interactions were parsed into genotype effects at each individual age. Statistical p-values are shown in Supplementary Tables S1 and S4.

## Supporting Information

Table S1 Affymetrix Probe Sets and Annotations with Significant Genotype and Age Effects FDR<0.10
(0.12 MB DOC)

Table S2 DAVID Complete Functional Annotation Clusters
(0.13 MB XLS)

Table S3 Summary of statistical main effects on quantitative PCR gene expression in paternal allele study.
(0.13 MB DOC)

Table S4 Least square means and standard errors of gene expression in semimembranosus of paternal allele study.
(0.19 MB DOC)

Table S5 Least square means and standard errors of gene expression in supraspinatus of paternal allele study.
(0.22 MB DOC)

Table S6 Summary of statistical main effects on qPCR gene expression in maternal allele study.
(0.05 MB DOC)

Table S7 Least square means and standard errors of gene expression in maternal allele study.
(0.17 MB DOC)

Table S8 Quantitative PCR primer sequences, amplification conditions, and sequence identities
(0.13 MB DOC)
