# Supplementary material for: Effect of DLK1 and RTL1 but Not MEG3 or MEG8 on Muscle Gene Expression in Callipyge Lambs
Source: PLoS One. 2009 Oct 9;4(10):e7399. doi: 10.1371/journal.pone.0007399 (PMC2756960; doi:10.1371/journal.pone.0007399)
Supplement: Table S3 — Summary of statistical main effects on quantitative PCR gene expression in paternal allele study. (0.13 MB DOC) [file pone.0007399.s003.doc]

|  | P-Values for Main Effects in Transcripts Validated by qPCR | | | | | | | | | |
| --- | --- | --- | --- | --- | --- | --- | --- | --- | --- | --- |
|  | *Semimembranosus* | | | *Longissimus dorsi* | | | *Supraspinatus* | | | |
| Gene | Geno^1^ | Age | AxG^2^ | Geno | Age | AxG | Geno | | Age | AxG |
| *APOD* | 0.0001 | 0.0017 | 0.0626 | <0.0001 | 0.0166 | 0.0366 | 0.0718 | | 0.0046 | 0.4887 |
| *AKR1C4* | <0.0001 | 0.0006 | 0.5933 | <0.0001 | <0.0001 | 0.2939 | not detectable | | | |
| *ATF4* | 0.3130 | <0.0001 | 0.7651 | 0.0043 | <0.0001 | 0.8319 | 0.6376 | | 0.0002 | 0.5607 |
| *BHLHB3* | 0.0027 | 0.0059 | 0.2454 | 0.0019 | 0.5905 | 0.8799 | 0.8141 | | 0.0070 | 0.8455 |
| *CABC1* | 0.0017 | 0.0394 | 0.2796 | 0.2161 | <0.0001 | 0.7810 | 0.0683 | | 0.2009 | 0.8667 |
| *CAST* | 0.0067 | 0.0055 | 0.5469 | 0.0794 | <0.0001 | 0.5831 | 0.5051 | | 0.1045 | 0.7138 |
| *CDO1* | <0.0001 | 0.0032 | 0.5923 | 0.0474 | <0.0001 | 0.7137 | 0.8501 | | 0.0041 | 0.6556 |
| *COQ10A* | 0.0058 | 0.2704 | 0.1683 | 0.0048 | 0.2698 | 0.4498 | 0.1890 | | 0.0001 | 0.3931 |
| *DLK1* | <0.0001 | 0.1297 | 0.0012 | <0.0001 | 0.5347 | 0.0019 | 0.5711 | | 0.4235 | 0.3289 |
| *DNTTIP1* | 0.0002 | <0.0001 | 0.0052 | <0.0001 | <0.0001 | 0.0262 | 0.3516 | | <0.0001 | 0.1365 |
| *FCGRT* | 0.0228 | <0.0001 | 0.6279 | 0.5566 | <0.0001 | 0.8514 | 0.5777 | | 0.0151 | 0.4489 |
| *HDAC9* | 0.0005 | 0.8057 | 0.0475 | not measured^3^ | | | 0.2336 | 0.8534 | | 0.3583 |
| *HIPK2* | 0.0368 | 0.0525 | 0.2270 | 0.0307 | 0.2009 | 0.5433 | 0.6769 | | 0.7954 | 0.4565 |
| *IDH2* | 0.0020 | 0.1898 | 0.8578 | 0.0034 | <0.0001 | 0.7220 | 0.5529 | | 0.2281 | 0.7866 |
| *KCNN3* | 0.0218 | 0.0014 | 0.0562 | 0.0019 | 0.0078 | 0.0209 | 0.0994 | | 0.0027 | 0.6903 |
| *LOC513822* | 0.0104 | <0.0001 | 0.7029 | 0.0282 | <0.0001 | 0.9955 | 0.5605 | | 0.0003 | 0.2541 |
| *LPL* | <0.0001 | 0.0053 | 0.6331 | 0.0068 | <0.0001 | 0.6205 | 0.5442 | | <0.0001 | 0.5984 |
| *MAPK6* | <0.0001 | 0.1057 | 0.4413 | 0.0044 | 0.0992 | 0.4028 | 0.8606 | | 0.0022 | 0.5012 |
| *MEG3* | 0.0028 | 0.5558 | 0.0514 | 0.0001 | 0.0002 | 0.2609 | 0.9125 | | 0.0192 | 0.1751 |
| *PARK7* | 0.0105 | <0.0001 | 0.2493 | <0.0001 | 0.0001 | 0.0591 | 0.7886 | | 0.4990 | 0.2624 |
| *PDE4D* | 0.0198 | <0.0001 | 0.3918 | 0.0016 | <0.0001 | 0.1164 | 0.8817 | | 0.0002 | 0.4358 |
| *PDE7A* | 0.0211 | <0.0001 | 0.5847 | <0.0001 | 0.2063 | 0.2693 | 0.0389 | | 0.2443 | 0.5009 |
| *PFKM* | 0.0002 | <0.0001 | 0.7359 | 0.0198 | <0.0001 | 0.9596 | 0.8790 | | 0.8133 | 0.8062 |
| *RPS6KA3* | 0.6769 | 0.0003 | 0.0099 | 0.0032 | 0.0913 | 0.6462 | 0.8868 | | 0.0278 | 0.6652 |
| *RSPRY1* | 0.0273 | 0.4107 | 0.0355 | 0.0328 | 0.2390 | 0.1864 | 0.7395 | | 0.1241 | 0.7528 |
| *SLC22A3* | <0.0001 | 0.0002 | <0.0001 | <0.0001 | 0.0192 | 0.0212 | 0.8826 | | 0.7446 | 0.2581 |
| *TXNIP* | 0.0002 | 0.0138 | 0.0181 | 0.5992 | 0.0007 | 0.3965 | 0.8191 | | 0.0145 | 0.8028 |
| *RTL1* | <0.0001 | 0.0678 | 0.0850 |  |  |  | <0.0001 | | 0.0340 | 0.0967 |
| *RPLP0-control* | 0.1454 | 0.0004 | 0.1654 | 0.4898 | 0.0256 | 0.4302 | 0.8500 | | 0.0005 | 0.6328 |

|  | P-Values for Main Effects in Transcripts Not Validated by qPCR | | | | | | | | |
| --- | --- | --- | --- | --- | --- | --- | --- | --- | --- |
|  | *Semimembranosus* | | | *Longissimus dorsi* | | | *Supraspinatus* | | |
| Gene | Geno^1^ | Age | AxG^2^ | Geno | Age | AxG | Geno | Age | AxG |
| *ARHGAP18* | 0.7623 | 0.1252 | 0.0044 | not measured | | | not measured | | |
| CB535183 | 0.7058 | 0.0014 | 0.0465 | not measured | | | not measured | | |
| *DTNA* | 0.5113^4^ |  |  | not measured | | | not measured | | |
| *ECH1* | 0.7695^4^ |  |  | not measured | | | not measured | | |
| *HMGN2* | 0.2192^4^ |  |  | not measured | | | not measured | | |
| *LOC510404* | 0.6250^4^ |  |  | not measured | | | not measured | | |
| *LOC789894* | 0.4600 | 0.0004 | 0.0218 | not measured | | | not measured | | |
| *MLYCD* | 0.2412 | 0.2212 | 0.9359 | not measured | | | not measured | | |
| *MYL3* | 0.1774^4^ |  |  | not measured | | | not measured | | |
| *NME4* | 0.7930^4^ |  |  | not measured | | | not measured | | |
| *PDLIM1* | 0.1607 | 0.0002 | 0.9922 | not measured | | | not measured | | |
| *PDLIM7* | 0.7070^4^ |  |  | not measured | | | not measured | | |
| *PFKFB1* | 0.2739 | <0.0001 | 0.8285 | not measured | | | not measured | | |
| *PGM1* | 0.3023^4^ |  |  | not measured | | | not measured | | |
| *PIAS2* | 0.7150^4^ |  |  | not measured | | | not measured | | |
| *PKM2* | 0.2174 | 0.0001 | 0.0322 | not measured | | | not measured | | |
| *ROCK2* | 0.6670 | 0.0022 | 0.2335 | not measured | | | not measured | | |
| *TCEA3* | 0.5907 | 0.0018 | 0.0024 | not measured | | | not measured | | |
| *TEX2* | 0.2029^4^ |  |  | not measured | | | not measured | | |
| *TRAF3IP3* | 0.2163 | 0.0455 | 0.1091 | not measured | | | not measured | | |
| *TTYH1* | 0.6681 | 0.1973 | 0.7010 | not measured | | | not measured | | |
| *UCHL1* | 0.6410^4^ |  |  | not measured | | | not measured | | |

^1^Effect of Genotype
^2^Age by genotype interaction effect

^3^Measured by Vuocolo et al.[48].

^4^No significant effect of genotype at one post-hypertrophy age, no additional ages assayed by qPCR.
