# Supplementary material for: Effect of DLK1 and RTL1 but Not MEG3 or MEG8 on Muscle Gene Expression in Callipyge Lambs
Source: PLoS One. 2009 Oct 9;4(10):e7399. doi: 10.1371/journal.pone.0007399 (PMC2756960; doi:10.1371/journal.pone.0007399)
Supplement: Table S4 — Least square means and standard errors of gene expression in semimembranosus of paternal allele study. (0.19 MB DOC) [file pone.0007399.s004.doc]

|  |  | Age | | | | | |
| --- | --- | --- | --- | --- | --- | --- | --- |
| Gene | Genotype | -14d | 10d | 30d | 60d | 110d | 150d |
| *APOD* | *+/+* | 3.799 ± 0.261 | 3.481 ± 0.223 | 4.100 ± 0.223 | 3.922 ± 0.213 | 4.356 ± 0.213 | 3.993 ± 0.193 |
|  | *+/CLPG* | 3.373 ± 0.213 | 4.457 ± 0.223 | 4.503 ± 0.223 | 4.077 ± 0.213 | 4.495 ± 0.213 | 4.453 ± 0.173 |
| *ARHGAP18* | *+/+* | 4.572 ± 0.217 | 4.164 ± 0.217 | 4.443 ± 0.217 | 4.067 ± 0.188 | 4.138 ± 0.217 | 4.344 ± 0.188 |
|  | *+/CLPG* | 3.329 ± 0.217 | 4.635 ± 0.217 | 4.660 ± 0.217 | 4.458 ± 0.188 | 4.337 ± 0.228 | 4.529 ± 0.217 |
| *ARK1C4*^2^ | *+/+* |  | 4.166 ± 0.167 | 4.263 ± 0.167 |  |  | 3.885 ± 0.151 |
|  | *+/CLPG* |  | 3.673 ± 0.167 | 2.942 ± 0.167 |  |  | 2.863 ± 0.130 |
| *ATF4* | *+/+* | 5.057 ± 0.182 | 5.697 ± 0.182 | 5.778 ± 0.182 | 5.894 ± 0.182 | 5.888 ± 0.182 | 5.660 ± 0.158 |
|  | *+/CLPG* | 4.816 ± 0.182 | 5.76 ± 0.182 | 6.001 ± 0.182 | 6.133 ± 0.182 | 6.125 ± 0.182 | 5.767 ± 0.141 |
| *BHLHB3* | *+/+* | 3.284 ± 0.171 | 3.665 ± 0.171 | 3.786 ± 0.171 | 3.517 ± 0.171 | 3.786 ± 0.171 | 4.081 ± 0.148 |
|  | *+/CLPG* | 3.019 ± 0.171 | 3.447 ± 0.171 | 3.037 ± 0.171 | 3.526 ± 0.171 | 3.654 ± 0.171 | 3.534 ± 0.132 |
| *CABC1* | *+/+* | 4.823 ± 0.144 | 4.901 ± 0.144 | 4.992 ± 0.144 | 4.721 ± 0.128 | 4.744 ± 0.144 | 5.173 ± 0.124 |
|  | *+/CLPG* | 4.217 ± 0.144 | 4.706 ± 0.144 | 4.617 ± 0.144 | 4.752 ± 0.124 | 4.602 ± 0.144 | 4.809 ± 0.124 |
| *CAST^2^* | *+/+* |  | 4.692 ± 0.111 | 4.855 ± 0.111 |  |  | 5.061 ± 0.096 |
|  | *+/CLPG* |  | 5.030 ± 0.118 | 4.934 ± 0.111 |  |  | 5.060 ± 0.090 |
| CB535183 | *+/+* | 5.292 ± 0.193 | 5.341 ± 0.193 | 5.284 ± 0.193 | 5.730 ± 0.173 | 5.426 ± 0.193 | 5.794 ± 0.167 |
|  | *+/CLPG* | 4.500 ± 0.193 | 5.765 ± 0.193 | 5.517 ± 0.193 | 5.690 ± 0.167 | 5.745 ± 0.193 | 5.737 ± 0.167 |
| *CDO1* | *+/+* | 3.551 ± 0.215 | 2.971 ± 0.215 | 2.490 ± 0.215 | 2.724 ± 0.186 | 2.563 ± 0.215 | 2.492 ± 0.186 |
|  | *+/CLPG* | 2.516 ± 0.215 | 2.521 ± 0.215 | 2.254 ± 0.215 | 2.202 ± 0.186 | 2.003 ± 0.215 | 1.888 ± 0.186 |
| *COQ10A^2^* | *+/+* |  | 4.431 ± 0.097 | 4.510 ± 0.097 |  |  | 4.666 ± 0.081 |
|  | *+/CLPG* |  | 4.893 ± 0.094 | 4.720 ± 0.094 |  |  | 4.699 ± 0.073 |
| *DLK1* | *+/+* | 2.764 ± 0.241 | 3.677 ± 0.247 | 3.081 ± 0.241 | 2.418 ± 0.241 | 2.757 ± 0.241 | 3.496 ± 0.209 |
|  | *+/CLPG* | 2.469 ± 0.241 | 4.696 ± 0.241 | 4.696 ± 0.247 | 3.814 ± 0.241 | 3.833 ± 0.241 | 4.912 ± 0.187 |
| *DNTTIP1* | *+/+* | 4.166 ± 0.131 | 4.067 ± 0.131 | 4.353 ± 0.131 | 4.731 ± 0.131 | 4.931 ± 0.131 | 5.122 ± 0.113 |
|  | *+/CLPG* | 3.788 ± 0.133 | 4.749 ± 0.131 | 4.960 ± 0.131 | 5.210 ± 0.131 | 5.180 ± 0.131 | 5.350 ± 0.101 |
| *FCGRT* | *+/+* | 4.398 ± 0.177 | 5.205 ± 0.177 | 4.882 ± 0.177 | 4.398 ± 0.153 | 4.480 ± 0.177 | 4.170 ± 0.153 |
|  | *+/CLPG* | 3.871 ± 0.194 | 4.864 ± 0.177 | 4.641 ± 0.177 | 4.333 ± 0.153 | 4.199 ± 0.177 | 4.200 ± 0.153 |
| *HDAC9* | *+/+* | 3.063 ± 0.171 | 2.755 ± 0.171 | 2.473 ± 0.171 | 2.697 ± 0.148 | 2.499 ± 0.171 | 2.775 ± 0.148 |
|  | *+/CLPG* | 2.017 ± 0.171 | 2.543 ± 0.171 | 2.414 ± 0.171 | 2.462 ± 0.148 | 2.425 ± 0.171 | 2.178 ± 0.148 |
| *HIPK2^2^* | *+/+* |  | 1.762 ± 0.161 | 2.111 ± 0.165 |  |  | 2.313 ± 0.140 |
|  | *+/CLPG* |  | 2.256 ± 0.161 | 2.147 ± 0.197 |  |  | 2.151 ± 0.125 |
| *IDH2* | *+/+* | 5.090 ± 0.158 | 5.264 ± 0.158 | 5.131 ± 0.158 | 5.012 ± 0.137 | 5.207 ± 0.158 | 5.028 ± 0.137 |
|  | *+/CLPG* | 4.618 ± 0.158 | 5.111 ± 0.158 | 4.670 ± 0.158 | 4.746 ± 0.137 | 5.009 ± 0.158 | 4.795 ± 0.137 |
| *KCNN3* | *+/+* | 3.601 ± 0.278 | 3.510 ± 0.278 | 3.997 ± 0.278 | 4.190 ± 0.278 | 4.307 ± 0.278 | 4.361 ± 0.241 |
|  | *+/CLPG* | 3.046 ± 0.278 | 4.911 ± 0.278 | 4.360 ± 0.278 | 4.566 ± 0.278 | 4.667 ± 0.278 | 4.696 ± 0.215 |
|  |  |  | | | | | |
|  |  |  | | | | | |
|  |  | Age | | | | | |
| Gene | Genotype | -14d | 10d | 30d | 60d | 110d | 150d |
| LOC513822 | *+/+* | 1.528 ± 0.300 | 4.185 ± 0.319 | 4.226 ± 0.319 | 4.383 ± 0.300 | 4.145 ± 0.300 | 4.065 ± 0.276 |
|  | *+/CLPG* | 2.138 ± 0.317 | 4.989 ± 0.319 | 4.723 ± 0.319 | 4.226 ± 0.300 | 4.911 ± 0.300 | 4.523 ± 0.247 |
| LOC789894 | *+/+* | 4.367 ± 0.137 | 4.428 ± 0.134 | 4.454 ± 0.134 | 4.682 ± 0.116 | 4.547 ± 0.134 | 4.588 ± 0.116 |
|  | *+/CLPG* | 3.790 ± 0.134 | 4.819 ± 0.134 | 4.622 ± 0.134 | 4.796 ± 0.116 | 4.797 ± 0.134 | 4.575 ± 0.116 |
| *LPL* | *+/+* | 3.269 ± 0.170 | 3.531 ± 0.170 | 3.859 ± 0.170 | 3.410 ± 1.147 | 3.885 ± 0.170 | 3.955 ± 0.147 |
|  | *+/CLPG* | 2.677 ± 0.170 | 3.252 ± 0.170 | 3.184 ± 0.170 | 3.078 ± 0.147 | 3.191 ± 0.170 | 3.267 ± 0.147 |
| *MAPK6^2^* | *+/+* |  | 4.902 ± 0.131 | 5.035 ± 0.131 |  |  | 5.057 ± 0.114 |
|  | *+/CLPG* |  | 5.260 ± 0.131 | 5.301 ± 0.140 |  |  | 5.292 ± 0.102 |
| *MEG3* | *+/+* | 6.986 ± 0.180 |  | 5.903 ± 0.243 | 6.151 ± 0.180 | 6.436 ± 0.180 |  |
|  | *+/CLPG* | 6.580 ± 0.180 |  | 6.757 ± 0.211 | 6.863 ± 0.180 | 6.811 ± 0.180 |  |
| *PARK7^2^* | *+/+* | 1.998 ± 0.239 | 2.526 ± 0.239 | 2.527 ± 0.239 | 2.573 ± 0.239 | 2.892 ± 0.239 | 2.451 ± 0.207 |
|  | *+/CLPG* | 1.713 ± 0.239 | 3.257 ± 0.239 | 2.834 ± 0.239 | 2.833 ± 0.239 | 3.324 ± 0.239 | 3.220 ± 0.185 |
| *PDE4D* | *+/+* | 2.362 ± 0.286 | 3.575 ± 0.286 | 3.810 ± 0.286 | 2.979 ± 0.286 | 3.326 ± 0.286 | 3.781 ± 0.248 |
|  | *+/CLPG* | 2.004 ± 0.286 | 4.284 ± 0.286 | 4.150 ± 0.286 | 3.840 ± 0.286 | 3.728 ± 0.286 | 4.219 ± 0.222 |
| *PDE7A* | *+/+* | 2.397 ± 0.301 | 3.671 ± 0.301 | 3.341 ± 0.304 | 2.961 ± 0.301 | 2.838 ± 0.301 | 3.785 ± 0.261 |
|  | *+/CLPG* | 2.214 ± 0.301 | 4.270 ± 0.301 | 4.270 ± 0.301 | 3.454 ± 0.301 | 3.071 ± 0.301 | 4.203 ± 0.233 |
| *PDLIM1* | *+/+* | 4.390 ± 0.204 | 4.987 ± 0.204 | 5.402 ± 0.204 | 5.406 ± 0.177 | 5.310 ± 0.204 | 5.041 ± 0.177 |
|  | *+/CLPG* | 4.203 ± 0.204 | 4.977 ± 0.204 | 5.214 ± 0.204 | 5.165 ± 0.177 | 5.080 ± 0.204 | 4.922 ± 0.177 |
| *PFKFB1* | *+/+* | 2.770 ± 0.162 | 3.605 ± 0.162 | 3.359 ± 0.162 | 3.498 ± 0.140 | 3.544 ± 0.162 | 3.866 ± 0.140 |
|  | *+/CLPG* | 2.725 ± 0.162 | 3.663 ± 0.162 | 3.539 ± 0.162 | 3.810 ± 0.140 | 3.667 ± 0.162 | 3.839 ± 0.140 |
| *PFKM* | *+/+* | 2.088 ± 0.152 | 2.857 ± 0.152 | 3.276 ± 0.152 | 2.047 ± 0.152 | 2.316 ± 0.152 | 3.152 ± 0.131 |
|  | *+/CLPG* | 2.015 ± 0.262 | 3.374 ± 0.152 | 3.359 ± 0.152 | 2.648 ± 0.152 | 2.613 ± 0.152 | 3.483 ± 0.117 |
| *PKM2* | *+/+* | 6.315 ± 0.072 | 6.454 ± 0.072 | 6.512 ± 0.072 | 6.349 ± 0.062 | 6.381 ± 0.072 | 6.452 ± 0.062 |
|  | *+/CLPG* | 6.050 ± 0.072 | 6.627 ± 0.072 | 6.527 ± 0.072 | 6.482 ± 0.062 | 6.440 ± 0.072 | 6.637 ± 0.062 |
| *ROCK2* | *+/+* | 3.774 ± 0.225 | 4.207 ± 0.225 | 3.854 ± 0.225 | 4.020 ± 0.195 | 4.244 ± 0.225 | 4.208 ± 0.195 |
|  | *+/CLPG* | 3.090 ± 0.225 | 4.533 ± 0.225 | 4.001 ± 0.225 | 4.352 ± 0.195 | 4.304 ± 0.225 | 4.351 ± 0.195 |
| *RPS6K* | *+/+* | 5.333 ± 0.197 | 5.423 ± 0.197 | 5.738 ± 0.197 | 5.290 ± 0.170 | 5.071 ± 0.197 | 5.582 ± 0.170 |
|  | *+/CLPG* | 4.221 ± 0.197 | 5.703 ± 0.203 | 5.753 ± 0.197 | 5.568 ± 0.170 | 5.261 ± 0.197 | 5.656 ± 0.170 |
| *RSPRY1*^2^ | *+/+* |  | 4.487 ± 0.086 | 4.643 ± 0.088 |  |  | 4.593 ± 0.075 |
|  | *+/CLPG* |  | 4.712 ± 0.086 | 4.421 ± 0.088 |  |  | 4.656 ± 0.067 |
| *SLC22A* | *+/+* | 2.209 ± 0.185 | 2.310 ± 0.180 | 2.340 ± 0.180 | 2.629 ± 0.170 | 1.759 ± 0.185 | 2.033 ± 0.159 |
|  | *+/CLPG* | 1.751 ± 0.185 | 3.107 ± 0.185 | 3.171 ± 0.180 | 3.276 ± 0.170 | 3.256 ± 0.185 | 3.492 ± 0.156 |
| *TRAF3IP3* | *+/+* | 2.543 ± 0.207 | 2.750 ± 0.207 | 2.488 ± 0.207 | 2.666 ± 0.179 | 2.422 ± 0.207 | 2.582 ± 0.179 |
|  | *+/CLPG* | 1.907 ± 0.207 | 3.168 ± 0.207 | 2.866 ± 0.207 | 2.700 ± 0.179 | 2.875 ± 0.207 | 2.803 ± 0.179 |
| *TXNIP* | *+/+* | 4.946 ± 0.143 | 4.723 ± 0.143 | 4.804 ± 0.143 | 4.979 ± 0.124 | 4.567 ± 0.143 | 4.941 ± 0.124 |
|  | *+/CLPG* | 4.016 ± 0.143 | 4.350 ± 0.143 | 4.442 ± 0.143 | 4.946 ± 0.124 | 4.666 ± 0.143 | 4.536 ± 0.124 |
|  |  | Age | | | | | |
| Gene | Genotype | -14d | 10d | 30d | 60d | 110d | 150d |
| *RPLP0* | *+/+* | 4.230 ± 0.159 | 4.463 ± 0.159 | 4.219 ± 0.159 | 4.351 ± 0.159 | 4.340 ± 0.159 | 4.366 ± 0.137 |
|  | *+/CLPG* | 3.719 ± 0.159 | 4.611 ± 0.159 | 4.139 ± 0.162 | 4.320 ± 0.159 | 4.306 ± 0.159 | 4.258 ± 0.123 |
| *RTL1* | *+/+* | 4.209 ± 0.311 | 3.342 ± 0.442 (20d) | 3.405 ± 0.622 | 2.346 ± 0.442 (45d) | 2.324 ± 0.628 (60d) |  |
|  | *+/CLPG* | 4.432 ± 0.359 | 4.516 ± 0.311 (20d) | 5.244 ± 0.359 | 4.673 ± 0.311 (45d) | 4.056 ± 0.311 (60d) |  |

^1^Log10 of least square means and standard errors for transcript abundance per 100 ng input RNA.

^2^All transcripts were measured on at least six different age points to calculate effect of genotype. Other ages measured to obtain data for these transcripts were 20, 45, 80, 90, 130 and 200 days of age (data not shown in table).
