# Supplementary material for: Effect of DLK1 and RTL1 but Not MEG3 or MEG8 on Muscle Gene Expression in Callipyge Lambs
Source: PLoS One. 2009 Oct 9;4(10):e7399. doi: 10.1371/journal.pone.0007399 (PMC2756960; doi:10.1371/journal.pone.0007399)
Supplement: Table S5 — Least square means and standard errors of gene expression in supraspinatus of paternal allele study. (0.22 MB DOC) [file pone.0007399.s005.doc]

|  |  | Age | | | | | |
| --- | --- | --- | --- | --- | --- | --- | --- |
| Gene | Genotype | -14d | 10d | 30d | 60d | 110d | 150d |
| *APOD* | *+/+* | 1.869 ± 0.229 | 2.340 ± 0.229 | 2.480 ± 0.229 | 2.280 ± 0.229 | 1.515 ± 0.229 | 2.572 ± 0.198 |
|  | *+/CLPG* | 1.991 ± 0.229 | 2.369 ± 0.229 | 2.427 ± 0.280 | 2.283 ± 0.229 | 2.453 ± 0.229 | 2.555 ± 0.177 |
| *ARHGAP18* | *+/+* | not measured |  |  |  |  |  |
|  | *+/CLPG* |  |  |  |  |  |  |
| *AKR1C4* | *+/+* | Not detectable |  |  |  |  |  |
|  | *+/CLPG* |  |  |  |  |  |  |
| *ATF4* | *+/+* | 5.190 ± 0.130 | 5.212 ± 0.130 | 5.400 ± 0.130 | 5.522 ± 0.130 | 5.216 ± 0.130 | 5.487 ± 0.113 |
|  | *+/CLPG* | 5.387 ± 0.130 | 5.375 ± 0.130 | 5.378 ± 0.130 | 5.444 ± 0.130 | 5.336 ± 0.130 | 5.468 ± 0.101 |
| *BHLHB3* | *+/+* | 2.605 ± 0.199 | 3.379 ± 0.195 | 3.382 ± 0.195 | 3.408 ± 0.195 | 3.235 ± 0.195 | 3.572 ± 0.169 |
|  | *+/CLPG* | 2.835 ± 0.195 | 3.288 ± 0.195 | 3.311 ± 0.239 | 3.172 ± 0.195 | 3.370 ± 0.195 | 3.450 ± 0.151 |
| *CABC1* | *+/+* | 4.466 ± 0.177 | 5.079 ± 0.177 | 4.913 ± 0.177 | 4.998 ± 0.153 | 4.877 ± 0.177 | 4.851 ± 0.153 |
|  | *+/CLPG* | 4.903 ± 0.177 | 5.065 ± 0.177 | 5.057 ± 0.177 | 5.216 ± 0.153 | 5.092 ± 0.177 | 4.962 ± 0.153 |
| *CAST^2^* | *+/+* |  | 3.730 ± 0.178 | 4.201 ± 0.178 |  |  | 3.875 ± 0.154 |
|  | *+/CLPG* |  | 4.009 ± 0.178 | 4.010 ± 0.178 |  |  | 4.082 ± 0.138 |
| CB535183 | *+/+* | not measured |  |  |  |  |  |
|  | *+/CLPG* |  |  |  |  |  |  |
| *CDO1* | *+/+* | 3.154 ± 0.187 | 2.871 ± 0.187 | 2.505 ± 0.187 | 2.795 ± 0.162 | 2.309 ± 0.187 | 2.626 ± 0.162 |
|  | *+/CLPG* | 3.141 ± 0.187 | 2.897 ± 0.187 | 2.739 ± 0.187 | 2.488 ± 0.187 | 2.435 ± 0.187 | 2.442 ± 0.162 |
| *COQ10A* | *+/+* | 4.158 ± 0.247 | 4.974 ± 0.226 | 5.577 ± 0.226 | 4.971± 0.195 | 4.877 ± 0.226 | 5.332 ± 0.195 |
|  | *+/CLPG* | 4.212 ± 0.247 | 5.363 ± 0.226 | 5.363 ± 0.226 | 4.969 ± 0.195 | 5.589 ± 0.226 | 5.419 ± 0.195 |
| *DLK1* | *+/+* | 5.193 ± 0.252 | 4.533 ± 0.252 | 4.327 ± 0.252 | 4.697 ± 0.252 | 4.386 ± 0.252 | 4.502 ± 0.218 |
|  | *+/CLPG* | 4.699 ± 0.252 | 4.689 ± 0.252 | 4.540 ± 0.309 | 4.292 ± 0.252 | 4.663 ± 0.252 | 4.644 ± 0.195 |
| *DNTTIP1* | *+/+* | 3.394 ± 0.158 | 3.906 ± 0.158 | 3.960 ± 0.158 | 4.122 ± 0.158 | 3.744 ± 0.158 | 4.360 ± 0.137 |
|  | *+/CLPG* | 3.134 ± 0.163 | 4.177 ± 0.158 | 4.202 ± 0.194 | 3.778 ± 0.158 | 4.136 ± 0.158 | 4.564 ± 0.125 |
| *FCGRT* | *+/+* | 3.936 ± 0.180 | 4.356 ± 0.180 | 4.526 ± 0.180 | 3.997 ± 0.156 | 3.878 ± 0.180 | 4.219 ± 0.156 |
|  | *+/CLPG* | 3.921 ± 0.180 | 4.494 ± 0.180 | 4.512 ± 0.180 | 4.319 ± 0.156 | 4.095 ± 0.180 | 3.980 ± 0.156 |
| *HDAC9* | *+/+* | 3.759 ± 0.146 | 3.897 ± 0.146 | 3.513 ± 0.146 | 3.836 ± 0.126 | 3.663 ± 0.146 | 3.581 ± 0.126 |
|  | *+/CLPG* | 4.380 ± 0.146 | 3.540 ± 0.146 | 3.729 ± 0.146 | 3.634 ± 0.126 | 3.678 ± 0.146 | 3.600 ± 0.126 |
| *HIPK2* | *+/+* | 3.643 ± 0.129 | 3.784 ± 0.129 | 3.793 ± 0.129 | 3.732 ± 0.112 | 3.534 ± 0.129 | 3.715 ± 0.112 |
|  | *+/CLPG* | 3.701 ± 0.129 | 3.592 ± 0.129 | 3.691 ± 0.129 | 3.505 ± 0.112 | 3.728 ± 0.129 | 3.803 ± 0.112 |
| *IDH2* | *+/+* | 4.646 ± 0.210 | 4.723 ± 0.210 | 4.318 ± 0.210 | 4.445 ± 0.182 | 4.594 ± 0.210 | 4.883 ± 0.182 |
|  | *+/CLPG* | 4.660 ± 0.210 | 4.789 ± 0.210 | 4.518 ± 0.210 | 4.263 ± 0.182 | 4.397 ± 0.210 | 4.563 ± 0.182 |
| *KCNN3* | *+/+* | 3.937 ± 0.175 | 4.166 ± 0.\175 | 4.202 ± 0.175 | 4.945 ± 0.175 | 4.488 ± 0.175 | 4.315 ± 0.152 |
|  | *+/CLPG* | 4.416 ± 0.175 | 4.304 ± 0.175 | 4.433 ± 0.214 | 4.861 ± 0.175 | 4.709 ± 0.175 | 4.361 ± 0.136 |
|  |  |  | | | | | |
|  |  |  | | | | | |
|  |  |  | | | | | |
|  |  | Age | | | | | |
| Gene | Genotype | -14d | 10d | 30d | 60d | 110d | 150d |
| LOC513822 | *+/+* | 3.348 ± 0.239 | 4.102 ± 0.195 | 3.530 ± 0.195 | 4.553 ± 0.195 | 3.889 ± 0.195 | 4.246 ± 0.169 |
|  | *+/CLPG* | 3.218 ± 0.195 | 4.148 ± 0.195 | 4.083 ± 0.239 | 4.158 ± 0.195 | 4.258 ± 0.195 | 4.208 ± 0.151 |
| LOC789894 | *+/+* | not measured |  |  |  |  |  |
|  | *+/CLPG* |  |  |  |  |  |  |
| *LPL* | *+/+* | 3.877 ± 0.188 | 4.832 ± 0.188 | 5.271 ± 0.188 | 5.310 ± 0.163 | 5.476 ± 0.188 | 4.999 ± 0.163 |
|  | *+/CLPG* | 3.961 ± 0.188 | 5.253 ± 0.188 | 5.311 ± 0.188 | 5.073 ± 0.163 | 5.436 ± 0.188 | 5.115 ± 0.163 |
| *MAPK6^2^* | *+/+* |  | 4.097 ± 0.166 | 4.276 ± 0.166 |  |  | 4.099 ± 0.144 |
|  | *+/CLPG* |  | 4.126 ± 0.166 | 4.026 ± 0.204 |  |  | 4.190 ± 0.129 |
| *MEG3^2^* | *+/+* | 6.895 ± 0.149 |  | 5.122 ± 0.231 | 6.634 ± 0.149 | 6.440 ± 0.149 |  |
|  | *+/CLPG* | 6.825 ± 0.149 |  | 5.709 ± 0.231 | 6.238 ± 0.149 | 6.476 ± 0.148 |  |
| *PARK7* | *+/+* | 1.823 ± 0.144 |  |  | 2.221 ± 0.144 | 2.104 ± 0.144 |  |
|  | *+/CLPG* | 1.861 ± 0.144 |  |  | 1.904 ± 0.144 | 2.039 ± 0.144 |  |
| *PDE4D* | *+/+* | 2.600 ± 0.336 | 3.669 ± 0.274 | 3.806 ± 0.274 | 4.484 ± 0.274 | 3.446 ± 0.274 | 3.724 ± 0.237 |
|  | *+/CLPG* | 2.398 ± 0.274 | 3.733 ± 0.274 | 3.784 ± 0.336 | 3.867 ± 0.274 | 4.023 ± 0.274 | 3.780 ± 0.212 |
| *PDE7A* | *+/+* | 3.069 ± 0.174 | 3.239 ± 0.164 | 3.208 ± 0.164 | 3.458 ± 0.164 | 3.474 ± 0.164 | 3.244 ± 0.142 |
|  | *+/CLPG* | 3.15 ± 0.164 | 3.580 ± 0.164 | 3.594 ± 0.201 | 3.373 ± 0.164 | 3.545 ± 0.164 | 3.683 ± 0.127 |
| *PDLIM1* | *+/+* | not measured |  |  |  |  |  |
|  | *+/CLPG* |  |  |  |  |  |  |
| *PFKFB1* | *+/+* | not measured |  |  |  |  |  |
|  | *+/CLPG* |  |  |  |  |  |  |
| *PFKM* | *+/+* | 1.237 ± 0.258 | 1.632 ± 0.240 | 1.556 ± 0.240 | 1.258 ± 0.258 | 0.972 ± 0.258 | 1.635 ± 0.208 |
|  | *+/CLPG* | 0.846 ± 0.258 | 1.503 ± 0.240 | 1.664 ± 0.294 | 1.334 ± 0.258 | 1.128 ± 0.258 | 1.527 ± 0.186 |
| *PKM2* | *+/+* | note measured |  |  |  |  |  |
|  | *+/CLPG* |  |  |  |  |  |  |
| *ROCK2* | *+/+* | not measured |  |  |  |  |  |
|  | *+/CLPG* |  |  |  |  |  |  |
| *RSPRY1* | *+/+* | 3.396 ± 0.092 | 3.119 ± 0.092 | 3.273 ± 0.092 | 3.359 ± 0.080 | 3.275 ± 0.092 | 3.266 ± 0.080 |
|  | *+/CLPG* | 3.445 ± 0.092 | 3.192 ± 0.092 | 3.168 ± 0.092 | 3.222 ± 0.080 | 3.332 ± 0.092 | 3.225 ± 0.092 |
| *RPS6K* | *+/+* | 4.917 ± 0.197 | 4.956 ± 0.197 | 5.087 ± 0.197 | 5.350 ± 0.171 | 5.192 ± 0.197 | 5.267 ± 0.171 |
|  | *+/CLPG* | 4.496 ± 0.197 | 5.173 ± 0.197 | 5.102 ± 0.197 | 5.390 ± 0.197 | 5.330 ± 0.197 | 5.185 ± 0.171 |
| *SLC22A* | *+/+* | 3.103 0.224 | 2.576 0.224 | 3.280 0.224 | 3.166 0.194 | 3.030 0.224 | 2.973 0.194 |
|  | *+/CLPG* | 2.990 0.224 | 3.228 0.232 | 3.164 0.224 | 2.765 0.194 | 2.816 0.224 | 3.053 0.194 |
| *TRAF3IP3* | *+/+* | not measured |  |  |  |  |  |
|  | *+/CLPG* |  |  |  |  |  |  |
|  |  |  |  |  |  |  |  |
|  |  |  |  |  |  |  |  |
|  |  |  |  |  |  |  |  |
|  |  | Age | | | | | |
| Gene | Genotype | -14d | 10d | 30d | 60d | 110d | 150d |
| *TXNIP* | *+/+* | 4.510 ± 0.143 | 4.090 ± 0.143 | 4.160 ± 0.143 | 4.590 ± 0.124 | 4.311 ± 0.143 | 4.448 ± 0.124 |
|  | *+/CLPG* | 4.320 ± 0.143 | 4.040 ± 0.143 | 4.340 ± 0.143 | 4.604 ± 0.124 | 4.375 ± 0.143 | 4.319 ± 0.124 |
| *RPLP0* | *+/+* | 5.351 ± 0.117 | 5.387 ± 0.117 | 5.214 ± 0.117 | 5.512 ± 0.117 | 5.293 ± 0.117 | 5.371 ± 0.102 |
|  | *+/CLPG* | 5.235 ± 0.117 | 5.298 ± 0.117 | 5.290 ± 0.144 | 5.317 ± 0.117 | 5.319 ± 0.117 | 5.273 ± 0.091 |
| *RTL1* | *+/+* | 1.827 ± 0.328 | 1.271 ± 0.379 (20d) | 0.640 ± 0.464 | 1.882 ± 0.330 (45d) | 1.971 ± 0.328 (60d) |  |
|  | *+/CLPG* | 1.970 ± 0.379 | 3.216 ± 0.328 (20d) | 2.138 ± 0.379 | 3.398 ± 0.328 (45d) | 2.580 ± 0.328 (60d) |  |

^1^Log10 of least square means and standard errors for transcript abundance per 100 ng input RNA.

^2^All transcripts were measured on at least six different age points to calculate effect of genotype. Other ages measured to obtain data for these transcripts were 20, 45, 80, 90, 130 and 200 days of age (data not shown in table).
