# Supplementary material for: Effect of DLK1 and RTL1 but Not MEG3 or MEG8 on Muscle Gene Expression in Callipyge Lambs
Source: PLoS One. 2009 Oct 9;4(10):e7399. doi: 10.1371/journal.pone.0007399 (PMC2756960; doi:10.1371/journal.pone.0007399)
Supplement: Table S6 — Summary of statistical main effects on qPCR gene expression in maternal allele study. (0.05 MB DOC) [file pone.0007399.s006.doc]

|  | P-Values for Main Effects in Transcripts Validated by qPCR | | | | | |
| --- | --- | --- | --- | --- | --- | --- |
|  | *Semimembranosus* | | | *Supraspinatus* | | |
| Gene | Geno^1^ | Age | AxG^2^ | Geno | Age | AxG |
| *EIF4A3* | 0.9229 | 0.3322 | 0.3173 | n/a |  |  |
| *MEG3^3^* | <0.0001 | 0.0037 | 0.0256 | <0.0001 | <0.0001 | 0.6708 |
| *MEG8^3^* | <0.0001 | 0.2855 | 0.0499 | <0.0001 | <0.0001 | 0.9722 |
| CB439344 | <0.0001 | 0.5829 | 0.5664 | 0.0003 | <0.0001 | 0.0077 |
| *PARK7* | 0.0328 | 0.0005 | 0.0591 | see Table C.5 | | |
| *PHKA* | 0.5994 | <0.0001 | 0.7037 | n/a |  |  |
| *PRPF3* | 0.2319 | 0.0493 | 0.4406 | n/a |  |  |
| *SEPHS2* | 0.1459 | 0.0158 | 0.2269 | n/a |  |  |
| *SP140* | 0.7271 | 0.0206 | 0.4846 | n/a |  |  |
| *TCEA3* | 0.8839 | <0.0001 | 0.4928 | n/a |  |  |
| *RPLP0* | 0.5236 | 0.3990 | 0.7085 | 0.8792 | 0.0066 | 0.0062 |

^1^Effect of Genotype
^2^Age by genotype interaction effect

^3^Quantitative PCR assays are based on ovine sequences from AF354168 and have been published [20].
