# Supplementary material for: Effect of DLK1 and RTL1 but Not MEG3 or MEG8 on Muscle Gene Expression in Callipyge Lambs
Source: PLoS One. 2009 Oct 9;4(10):e7399. doi: 10.1371/journal.pone.0007399 (PMC2756960; doi:10.1371/journal.pone.0007399)
Supplement: Table S7 — Least square means and standard errors of gene expression in maternal allele study. (0.17 MB DOC) [file pone.0007399.s007.doc]

|  |  |  | Age | | | | | | | | |
| --- | --- | --- | --- | --- | --- | --- | --- | --- | --- | --- | --- |
| Gene | Muscle | Genotype | -14 | | 20 | | 30 | | 45 | | 60 |
| *EIF4A3* | SM | +/+ | 4.0263 ± 0.141 | | 3.558 ± 0.182 | | 3.6285 ± 0.182 | |  | |  |
|  |  | C/+ | 3.8764 ± 0.1287 | | 3.5675 ± 0.1576 | | 3.5874 ± 0.1576 | |  | |  |
|  |  | +/C | 3.4945 ± 0.182 | | 3.9895 ± 0.3152 | | 3.604 ± 0.182 | |  | |  |
|  |  | C/C | 3.9283 ± 0.1576 | | 3.456 ± 0.3152 | | 3.9423 ± 0.182 | |  | |  |
|  | SS |  | not measured | |  | |  | |  | |  |
| *MEG3* | SM | +/+ | 7.7030 ± 0.1955 | | 6.5507 ± 0.2294 | | 6.7088 ± 0.2258 | | 6.6494 ± 0.1955 | | 6.595 ± 0.1955 |
|  |  | C/+ | 8.5257 ± 0.1596 | | 8.1481 ± 0.1955 | | 8.3624 ± 0.1955 | | 8.2395 ± 0.1955 | | 7.8678 ± 0.1955 |
|  |  | +/C | 6.9715 ± 0.2258 | | 6.9779 ± 0.1955 | | 7.1525 ± 0.2258 | | 7.7472 ± 0.1955 | | 7.3512 ± 0.1955 |
|  |  | C/C | 8.5082 ± 0.1955 | | 8.0548 ± 0.2258 | | 8.211 ± 0.2258 | | 8.4121 ± 0.1955 | | 8.0159 ± 0.1955 |
|  | SS | +/+ | 6.5379 ± 0.2 | | 5.3815 ± 0.2581 | | 5.6833 ± 0.2581 | | 5.2371 ± 0.2236 | | 5.3749 ± 0.2236 |
|  |  | C/+ | 7.2958 ± 0.1825 | | 6.453 ± 0.2236 | | 6.8253 ± 0.2236 | | 5.9631 ± 0.2236 | | 5.9031 ± 0.2236 |
|  |  | +/C | 6.3718 ± 0.2581 | | 5.5584 ± 0.2236 | | 5.4015 ± 0.2581 | | 5.5035 ± 0.2236 | | 5.3384 ± 0.2236 |
|  |  | C/C | 7.6203 ± 0.2236 | | 6.5077 ± 0.2581 | | 6.3318 ± 0.2581 | | 5.9901 ± 0.2236 | | 5.9506 ± 0.2269 |
| *MEG8* | SM | +/+ | 1.8637 ± 0.1821 | | 0.9125 ± 0.2351 | | 1.1555 ± 0.2351 | | 0.9711 ± 0.2036 | | 1.1751 ± 0.2036 |
|  |  | C/+ | 2.5747 ± 0.1662 | | 2.3818 ± 0.2036 | | 2.7311 ± 0.2036 | | 2.4905 ± 0.2036 | | 2.3287 ± 0.2036 |
|  |  | +/C | 1.2285 ± 0.2351 | | 1.3912 ± 0.2036 | | 1.522 ± 0.2351 | | 2.0989 ± 0.2036 | | 1.7145 ± 0.2036 |
|  |  | C/C | 2.7684 ± 0.2036 | | 2.4587 ± 0.2351 | | 2.473 ± 0.2351 | | 2.4141 ± 0.2036 | | 2.405 ± 0.2036 |
|  | SS | +/+ | 2.5928 ± 0.1403 | | 1.7485 ± 0.1811 | | 2.0917 ± 0.1811 | | 1.1802 ± 0.1568 | | 1.3849 ± 0.1568 |
|  |  | C/+ | 3.0485 ± 0.128 | | 2.4974 ± 0.1568 | | 2.727 ± 0.1568 | | 1.8339 ± 0.1568 | | 1.8313 ± 0.1568 |
|  |  | +/C | 2.4562 ± 0.1811 | | 1.7193 ± 0.1568 | | 1.9648 ± 0.1811 | | 1.3906 ± 0.1568 | | 1.2589 ± 0.1568 |
|  |  | C/C | 3.1309 ± 0.1568 | | 2.451 ± 0.1811 | | 2.5327 ± 0.1811 | | 2.0204 ± 0.1568 | | 1.9158 ± 0.1568 |
| *CB439344* | SM | +/+ | 2.2138 ± 0.2186 | | 1.465 ± 0.2822 | | 1.8478 ± 0.2822 | | 1.7273 ± 0.2444 | | 1.5755 ± 0.2444 |
|  |  | C/+ | 3.3143 ± 0.1995 | | 2.9119 ± 0.2444 | | 3.51 ± 0.2444 | | 3.0099 ± 0.2444 | | 2.9356 ± 0.2444 |
|  |  | +/C | 2.1193 ± 0.2822 | | 2.4856 ± 0.2444 | | 2.2003 ± 0.2822 | | 2.5703 ± 0.2444 | | 2.4236 ± 0.2492 |
|  |  | C/C | 3.4217 ± 0.2492 | | 3.265 ± 0.2822 | | 2.974 ± 0.2822 | | 3.1661 ± 0.2444 | | 3.0749 ± 0.2444 |
|  | SS | +/+ | 5.0129 ± 0.1362 | | 4.7072 ± 0.1742 | | 4.787 ± 0.1742 | | 3.349 ± 0.224 | | 3.402 ± 0.224 |
|  |  | C/+ | 5.2854 ± 0.1232 | | 5.0185 ± 0.1508 | | 5.0877 ± 0.1508 | | 3.6432 ± 0.1526 | | 3.7069 ± 0.1769 |
|  |  | +/C | 5.1008 ± 0.1742 | | 3.6422 ± 0.1584 | | 4.7383 ± 0.1742 | | 3.4613 ± 0.1798 | |  |
|  |  | C/C | 5.3928 ± 0.1508 | | 4.2225 ± 0.1742 | | 4.9874 ± 0.1769 | | 3.6742 ± 0.1526 | | 3.7877 ± 0.1526 |
| *PARK7* | SM | +/+ | 3.3706 ± 0.128 | | 3.4113 ± 0.1653 | | 3.7027 ± 0.1653 | | 3.6212 ± 0.1431 | | 3.3961 ± 0.1431 |
|  |  | C/+ | 3.4724 ± 0.1169 | | 3.4601 ± 0.1431 | | 3.5921 ± 0.1431 | | 3.6161 ± 0.1431 | | 3.7007 ± 0.1431 |
|  |  | +/C | 3.0417 ± 0.1653 | | 3.9905 ± 0.1431 | | 3.9447 ± 0.1653 | | 4.1603 ± 0.1431 | | 3.6947 ± 0.1458 |
|  |  | C/C | 3.5782 ± 0.1431 | | 3.7325 ± 0.1653 | | 3.8748 ± 0.1653 | | 3.7728 ± 0.1431 | | 3.47 ± 0.1458 |
|  | SS |  | not measured | |  | |  | |  | |  |
| *PHKA* | SM | +/+ | 2.7319 ± 0.1402 | | 3.0583 ± 0.1793 | | 3.414 ± 0.1793 | | 3.2639 ± 0.1552 | | 3.1511 ± 0.1552 |
|  |  | C/+ | 2.7755 ± 0.1268 | | 3.0889 ± 0.1552 | | 3.3518 ± 0.1552 | | 3.1276 ± 0.1552 | | 3.5175 ± 0.1552 |
|  |  | +/C | 2.5068 ± 0.1793 | | 3.2534 ± 0.1552 | | 3.4962 ± 0.1793 | | 3.5048 ± 0.1552 | | 3.4491 ± 0.1572 |
|  |  | C/C | 2.8285 ± 0.1552 | | 3.3192 ± 0.1793 | | 3.3738 ± 0.1793 | | 3.4136 ± 0.1552 | | 3.2770 ± 0.1552 |
|  | SS |  | not measured | |  | |  | |  | |  |
| *PRPF3* | SM | +/+ | 4.4306 ± 0.1686 | | 3.5313 ± 0.2176 | | 3.9228 ± 0.2176 | |  | |  |
|  |  | C/+ | 4.1335 ± 0.1539 | | 3.6205 ± 0.1885 | | 3.6134 ± 0.1885 | |  | |  |
|  |  | +/C | 3.8915 ± 0.2176 | | 3.836 ± 0.377 | | 4.012 ± 0.2176 | |  | |  |
|  |  | C/C | 4.2923 ± 0.1885 | | 4.16 ± 0.377 | | 4.1012 ± 0.2176 | |  | |  |
|  | SS |  | not measured | |  | |  | |  | |  |
|  |  |  |  | | | | | | | | |
|  |  |  |  | | | | | | | | |
|  |  |  |  | | | | | | | | |
|  |  |  |  | | | | | | | | |
|  |  |  |  | | | | | | | | |
|  |  |  |  |  | | Age | |  | |  | |
| Gene | Muscle | Genotype | -14 | 20 | | 30 | | 45 | | 60 | |
| *SEPHS2* | SM | +/+ | 3.3743 ± 0.136 | 3.2228 ± 0.1756 | | 3.447 ± 0.1756 | | 3.5216 ± 0.1521 | | 3.3259 ± 0.1521 | |
|  |  | C/+ | 3.4245 ± 0.1242 | 3.2889 ± 0.1521 | | 3.4595 ± 0.1521 | | 3.2681 ± 0.1521 | | 3.4302 ± 0.1521 | |
|  |  | +/C | 3.0478 ± 0.1756 | 3.1728 ± 0.1521 | | 3.4855 ± 0.1756 | | 3.4295 ± 0.1521 | | 3.1257 ± 0.1521 | |
|  |  | C/C | 3.4483 ± 0.1521 | 3.4597 ± 0.1756 | | 4.086 ± 0.1756 | | 3.4135 ± 0.1521 | | 3.0644 ± 0.1521 | |
|  | SS |  | not measured |  | |  | |  | |  | |
| *SP140* | SM | +/+ | 2.4661 ± 0.1842 | 1.701 ± 0.2353 | | 1.9135 ± 0.2353 | | 1.7674 ± 0.2038 | | 2.0107 ± 0.2038 | |
|  |  | C/+ | 2.1599 ± 0.1664 | 1.6832 ± 0.2038 | | 1.888 ± 0.2038 | | 1.9246 ± 0.2038 | | 1.8431 ± 0.2038 | |
|  |  | +/C | 1.7305 ± 0.2353 | 1.5771 ± 0.2038 | | 2.0127 ± 0.2353 | | 2.0364 ± 0.2038 | | 1.7443 ± 0.2065 | |
|  |  | C/C | 2.2501 ± 0.2038 | 1.804 ± 0.2353 | | 1.9703 ± 0.2353 | | 1.953 ± 0.2038 | | 1.3621 ± 0.2038 | |
|  | SS |  | not measured |  | |  | |  | |  | |
| *TCEA3* | SM | +/+ | 3.257 ± 0.4083 | 4.2912 ± 0.4619 | | 3.8422 ± 0.4445 | | 3.7434 ± 0.385 | | 4.89 ± 0.385 | |
|  |  | C/+ | 2.4784 ± 0.3607 | 4.485 ± 0.3961 | | 3.7549 ± 0.3961 | | 4.5589 ± 0.385 | | 4.3726 ± 0.385 | |
|  |  | +/C | 2.979 ± 0.5039 | 4.0188 ± 0.4445 | | 3.436 ± 0.4445 | | 4.6081 ± 0.385 | | 3.9242 ± 0.385 | |
|  |  | C/C | 3.2047 ± 0.4816 | 4.185 ± 0.4445 | | 3.6395 ± 0.4619 | | 4.5805 ± 0.385 | | 3.7914 ± 0.385 | |
|  | SS |  | not measured |  | |  | |  | |  | |
| *RPLP0* | SM | +/+ | 4.5974 ± 0.1899 | 4.6111 ± 0.254 | | 4.9087 ± 0.2452 | | 5.0043 ± 0.2123 | | 4.9946 ± 0.2123 | |
|  |  | C/+ | 5.0573 ± 0.1734 | 4.753 ± 0.2123 | | 4.9876 ± 0.2123 | | 4.858 ± 0.2123 | | 4.9368 ± 0.2123 | |
|  |  | +/C | 4.5813 ± 0.2452 | 4.5683 ± 0.2123 | | 4.8955 ± 0.2452 | | 4.9925 ± 0.2123 | | 4.6144 ± 0.218 | |
|  |  | C/C | 5.1138 ± 0.2123 | 4.8675 ± 0.2452 | | 5.0472 ± 0.2452 | | 4.9623 ± 0.2123 | | 4.514 ± 0.2123 | |
|  | SS | +/+ | 3.8351 ± 0.1054 | 3.7873 ± 0.1346 | | 3.7692 ± 0.1346 | | 4.2029 ± 0.1166 | | 4.2409 ± 0.1166 | |
|  |  | C/+ | 3.8573 ± 0.0952 | 3.7934 ± 0.1166 | | 3.9191 ± 0.1166 | | 3.9911 ± 0.1166 | | 4.0976 ± 0.1166 | |
|  |  | +/C | 3.6697 ± 0.1346 | 4.1694 ± 0.1166 | | 3.9195 ± 0.1346 | | 4.2088 ± 0.1166 | | 3.9952 ± 0.1166 | |
|  |  | C/C | 4.0745 ± 0.1166 | 3.9488 ± 0.1346 | | 3.9725 ± 0.1649 | | 4.2288 ± 0.1166 | | 3.5884 ± 0.1166 | |

^1^Log_10_ of least square means and standard errors for transcript abundance per 100 ng input RNA.
