# Supplementary material for: Effect of DLK1 and RTL1 but Not MEG3 or MEG8 on Muscle Gene Expression in Callipyge Lambs
Source: PLoS One. 2009 Oct 9;4(10):e7399. doi: 10.1371/journal.pone.0007399 (PMC2756960; doi:10.1371/journal.pone.0007399)
Supplement: Table S8 — Quantitative PCR primer sequences, amplification conditions, and sequence identities (0.13 MB DOC) [file pone.0007399.s008.doc]

| *Gene* | Primer Sequence | Anneal Temp | Capt. Temp | Prod. Size | Representative Public ID | Identity %^2^ |
| --- | --- | --- | --- | --- | --- | --- |
| *APOD* | F5’-TCTTGCTTTGCTTTTCCCCTATACC-3’  R5’-AGCTTGCCTTGGGTTCTTCTCC-3’ | 55˚C | 80˚C | 128 bp | [BC109863, DQ239921](http://www.ncbi.nlm.nih.gov/entrez/viewer.fcgi?db=nucleotide&val=81674720) | 97.2% |
| *ARHGAP18* | F5’-TATCAACTCAACCCCAATG-3’  R5’-TAATCTGGCAGCAGCAC-3’ | 48˚C | 80˚C | 221 bp | [XR_027470](http://www.ncbi.nlm.nih.gov/entrez/query.fcgi?cmd=Search&db=Nucleotide&term=XR_027470) | NA^3^ |
| *AKR1C4* | F5’-GGATCCCAACTATCATCAGA-3’  R5’-GGTTTGCTTGTGCTTTTT-3’ | 48˚C | 80˚C | 96 bp | [XM_581077](http://www.ncbi.nlm.nih.gov/entrez/viewer.fcgi?db=nucleotide&val=76650908) | 95.9% |
| *ATF4* | F5’-AGAGAGGAGTCAGGGCTCATA-3’  R5’-TGTCTTCCACTCCAGATCATTC-3’ | 52˚C | 51˚C | 178 bp | [NM_001034342](http://www.ncbi.nlm.nih.gov/entrez/viewer.fcgi?db=nucleotide&val=77735636) | 91.0% |
| *BHLHB3* | F5’-ATGTAAGGGGTGAGACACAACAGT-3’  R5’-CAAACAGTAGCAACAGCAGCAG-3’ | 54˚C | 80˚C | 99 bp | [XM_865112](http://www.ncbi.nlm.nih.gov/entrez/viewer.fcgi?db=nucleotide&val=76619292) | 99.5% |
| *CABC1* | F5’-GAGCTGAGCAGTGCCAACAAGTA-3’  R5’-TAGGAGGCGGCAGTTCTCTG-3’ | 57˚C | 84˚C | 120 bp | [NM_001046419](http://www.ncbi.nlm.nih.gov/entrez/query.fcgi?cmd=Search&db=Nucleotide&term=NM_001046419) | 95.0% |
| *CAST* | F5’- AGAGACCCACCAGGACGTG-3’  R5’- CAGGGGCTCTAGGCTAACA-3’ | 54˚C | 83˚C | 197 bp | NM_001030320 | 92.0% |
| *CDO1* | F5’- AAGGTCCAGCGAAGTTCC-3’  R5’- CAGGGGCTCTAGGCTAACA-3’ | 52˚C | 79˚C | 147 bp | NM_001034465 | 95.2% |
| CB439344 | F5’-CACTGAAAAGCACGTGCCTTCT-3’  R5’-GATTTAGCATCTGCCATGGACAA-3’ | 54˚C | 76˚C | 162 bp | [CB439344](http://www.ncbi.nlm.nih.gov/entrez/viewer.fcgi?db=nucleotide&val=29224258) | 90.4% |
| CB535183 | F5’-GGGAGGAGAGGTGAGTCA-3’  R5’-CTATTACAGGATTTGGGTGGTA-3’ | 50˚C | 78˚C | 157 bp | [CB535183](http://www.ncbi.nlm.nih.gov/entrez/query.fcgi?cmd=Search&db=Nucleotide&term=CB535183) | 96.2% |
| *DLK1* | F 5’-CCCGTCCTCTTGCTCCTGCT-3’ R 5’-GGCTGGCACCTGCACACACT-3’ | 58˚C | 82˚C | 116 bp | NM_174037 | NA^4^ |
| *DTNA* | F5’-GGTTACTTCAAATGGCGTCAA-3’  R5’-GGATTCCGCACAAACAACTC-3’ | 52˚C | 77˚C | 164 bp | [CK945807](http://www.ncbi.nlm.nih.gov/entrez/query.fcgi?cmd=Search&db=Nucleotide&term=CK945807) | NA^3^ |
| *ECH1* | F5’-CAAGAGACCTTCAGCGTCATC-3’  R5’-ATCTGCCGCCAAACCTAC-3’ | 53˚C | 85˚C | 168 bp | [XM_866888](http://www.ncbi.nlm.nih.gov/entrez/viewer.fcgi?db=nuccore&val=119910471) | NA^3^ |
| *EIF4A3* | F5’- CCGCATCCTCAGAGACAT-3’  F5’- ATAGATGAAGAGGCTCCAGTGA-3’ | 52˚C | 80˚C | 110 bp | NM_001046188 | 100% |
| *FCGRT* | F5’-TCGTCATCGGCTTATTCCT-3’  R5’-GATGATCGGCAGTTGCTG-3’ | 52˚C | 85˚C | 195 bp | [NM_176657](http://www.ncbi.nlm.nih.gov/entrez/query.fcgi?cmd=Search&db=Nucleotide&term=NM_176657) | 97.4% |
| *HMGN2* | F5’-CACAGACTTGACTCCCCTAACC-3’  R5’-TTTGAAGGACGCCATCTCA-3’ | 52˚C | 82˚C | 127 bp | [XM_589617](http://www.ncbi.nlm.nih.gov/entrez/viewer.fcgi?db=nucleotide&val=76611236) | NA^3^ |
| *IDH2* | F5’-CTGGACGCGTGGCCTAGAACA-3’  R5’-TTGCTGAGGCCGTGGATGC-3’ | 59˚C | 86˚C | 150 bp | [NM_175790](http://www.ncbi.nlm.nih.gov/entrez/query.fcgi?cmd=Search&db=Nucleotide&term=NM_175790) | 96.0% |
| *KCNN3* | F5’-CGGGAAACGTGGCTAATCTACA-3’  R5’-GTGTTGGCTTGGTCACTCAGCT-3’ | 55˚C | 83˚C | 152 bp | [XM_868686](http://www.ncbi.nlm.nih.gov/entrez/viewer.fcgi?db=nucleotide&val=76612085) | 97.0% |
| LOC789894 | F5’-CTCAGACAGGCGGGAAGAT-3’  R5’-TTTGGGTTGTACCTTGGAAGC-3’ | 54˚C | 83˚C | 126 bp | [XM_001256515](http://www.ncbi.nlm.nih.gov/entrez/viewer.fcgi?db=nuccore&val=119910012) | 98.4% |
| *LPL* | F5’-TCACGTATGAAGCCCCACAT-3’  R5’-AAGGAGTGTTCCGGCACCA-3’ | 55˚C | 80˚C | 119 bp | [NM_001075120](http://www.ncbi.nlm.nih.gov/entrez/query.fcgi?cmd=Search&db=Nucleotide&term=NM_001075120) | 90.0% |
| *MAPK6* | F5’-TCAAAGTCAGTAAGCCGAGAA-3’  R5’-AACAGTCCTCCCCACCAC-3’ | 52˚C | 80˚C | 115 bp | [XM_605323](http://www.ncbi.nlm.nih.gov/entrez/viewer.fcgi?db=nucleotide&val=76663342) | 97.9% |
|  |  |  |  |  |  |  |
|  |  |  |  |  |  |  |
| *Gene* | Primer Sequence | Anneal Temp | Capt. Temp | Prod. Size | Representative Public ID | Identity %^2^ |
| *TXNIP* | F5’-ATGTTGCCATGAGAAACCAGAA-3’  R5’-TAAGGCGGAGAGTGACTGACC-3’ | 52˚C | 84˚C | 140 bp | NM_001101875 | 97.9% |
| *MLYCD* | F5’-CCGTGGTCGCCTTAGC-3’  R5’-TTGTGTAGATTCGCAAACCTT-3’ | 51˚C | 78˚C | 111 bp | [XM_864349](http://www.ncbi.nlm.nih.gov/entrez/viewer.fcgi?db=nuccore&id=119909819) | 80.5% |
| *MYL3* | F5’-CATAGTTGATGCAGCCGTTGGA-3’  R5’-TGTTGAGGGTCTGCGTGTCTTC-3’ | 56˚C | 83˚C | 158 bp | [NM_001034342](http://www.ncbi.nlm.nih.gov/entrez/viewer.fcgi?db=nucleotide&val=77735636) | NA^4^ |
| *NME4* | F5’-CACCTCGAGGGCCATGATAG-3’  R5’-TGGATGACATTCCTGCTGACG-3’ | 55˚C | 84˚C | 98 bp | [NM_001077906](http://www.ncbi.nlm.nih.gov/entrez/query.fcgi?cmd=Search&db=Nucleotide&term=NM_001077906) | NA^3^ |
| *PARK7* | F5’-CGAGTCCGCTGCTGTGA-3’  R5’-TGACTTCCGTTCATCATTTTGT-3’ | 51˚C | 82˚C | 165 bp | [NM_001015572](http://www.ncbi.nlm.nih.gov/entrez/viewer.fcgi?db=nucleotide&val=62751848) | 100% |
| *PDE4D* | F5’-TAAACAGTACAAAGGGGACACT-3’  R5’-CTGTAAGAGAGGCTAAGAAACC-3’ | 50˚C | 75˚C | 151 bp | [AF536980](http://www.ncbi.nlm.nih.gov/entrez/viewer.fcgi?db=nucleotide&val=22901891) | 70.9% |
| *PDLIM1* | F5’-AAGCCCTCAGGATTCAGAAGT-3’  R5’-GAACACGCCGACAATGC-3’ | 53˚C | 84˚C | 117 bp | [NM_001035455](http://www.ncbi.nlm.nih.gov/entrez/viewer.fcgi?db=nuccore&val=78369681) | 96.6% |
| *PDLIM7* | F5’-GGGAGCGCCTTAGCCTTAG-3’  R5’-TGCTGGCATCTGGGACC-3’ | 56˚C | 86˚C | 213 bp | [NM_001017947](http://www.ncbi.nlm.nih.gov/entrez/viewer.fcgi?db=nuccore&id=62988301) | NA^3^ |
| *PHKA* | F5’-ATATTGCCAATGACTTGTTTCT-3’  R5’-TGCTTTGGAGAGGTAGGTC-3’ | 49˚C | 82˚C | 152 bp | [XM_609143.](http://www.ncbi.nlm.nih.gov/entrez/viewer.fcgi?db=nucleotide&val=76659298) | 100% |
| *PFKFB1* | F5’-CCTGGCCTACTTTCTGGACAA-3’  R5’-TTCTCAGGTTTCTCCCGATGT-3’ | 54˚C | 83˚C | 159 bp | [NM_174572](http://www.ncbi.nlm.nih.gov/entrez/viewer.fcgi?db=nuccore&val=94400820) | 97.5% |
| *PGM1* | F5’-TGGTTCTCGCATCATCTTTC-3’  R5’-ACCTTCAGGGCAATGGA-3’ | 51˚C | 84˚C | 150 bp | [NM_001076903](http://www.ncbi.nlm.nih.gov/entrez/query.fcgi?cmd=Search&db=Nucleotide&term=NM_001076903) | NA^3^ |
| *PKM2* | F5’-CTTGGGTCGGGTAGTTCA-3’  R5’-AATCGGGTATACAAAGGAAGG-3’ | 50˚C | 85˚C | 147 bp | [XM_590109](http://www.ncbi.nlm.nih.gov/entrez/viewer.fcgi?db=nuccore&id=119902009) | 93.2% |
| *PRPF3* | F5’- AACAGTGCCCTACAGAGAACA-3’  R5’- AAGGTAAGAGGGGAGAGGAAG-3’ | 53˚C | 83˚C | 143 bp | NM_001046051 | 97.9% |
| *ROCK2* | F5’-ACTGCGTCTTTAAGAAGTG-3’  R5’-ATTATGGCTTTATACAAGTGC-3’ | 47˚C | 77˚C | 195 bp | [NM_174452](http://www.ncbi.nlm.nih.gov/entrez/viewer.fcgi?db=nuccore&id=31341963) | 98.4% |
| *RPLP0* | F 5’-CAACCCTGAAGTGCTTGACAT-3’  R 5’-AGGCAGATGGATGGATCAGCCA-3’ | 50˚C | 84˚C | 208 bp | NM_001012682 | 100% |
| *SCL22A3* | F5’-TGAACAAAGGGAGAATGTAGGTA-3’  R5’-GCCTTAAAGCACATCAGCAT-3’ | 52˚C | 77˚C | 101 bp | CK968244 | 93.1% |
| *SEPHS2* | F5’- TGCACATTCCAAGGCCAGAA-3’  R5’- TCAATTAATCCCGTGCCAAGAAA-3’ | 52˚C | 81˚C | 167 bp | XR_028325 | 94.1% |
| *SP140* | F5’-GGCAAGCTGCAGTTTCACTCA-3’  R5’-GAATGCGGTCGGGTGCTAC-3’ | 57˚C | 81˚C | 161 bp | [BP103828](http://www.ncbi.nlm.nih.gov/entrez/viewer.fcgi?db=nucleotide&val=28306116) | 93.8% |
| *TCEA3* | F5’-CCTCCGTCTGCCTCTTG-3’  R5’-GTAATCATCATCCGCCTTCAG-3’ | 52˚C | 84˚C | 101 bp | [NM_001046360](http://www.ncbi.nlm.nih.gov/entrez/viewer.fcgi?db=nuccore&id=114052231) | 100% |
| *TEX2* | F5’-AAGTCATGTTCAGGCAAGGT-3’  R5’-AAGCTGCATTCAACATCAGA-3’ | 51˚C | 79˚C | 111 bp | NM_001101970 | 98.2% |
|  |  |  |  |  |  |  |
|  |  |  |  |  |  |  |
| *Gene* | Primer Sequence | Anneal Temp | Capt. Temp | Prod. Size | Representative Public ID | Identity %^2^ |
| *TRAF3IP3* | F5’-GCTGCAGGCCAGGATTGAAT-3’  R5’-TTGGTCACCAGGGCAAGTTTC-3’ | 56˚C | 83˚C | 117 bp | [BC119991](http://www.ncbi.nlm.nih.gov/entrez/viewer.fcgi?db=nuccore&val=111307011) | 98.3% |
| *TTYH1* | F5’-TTGCGTGGCCTGTGTGAAGA-3’  R5’-ACGAAGCGCTTGGATTCCTGA-3’ | 57˚C | 86˚C | 191 bp | [NM_001077015](http://www.ncbi.nlm.nih.gov/entrez/query.fcgi?cmd=Search&db=Nucleotide&term=NM_001077015) | 97.4% |
| *UCHL1* | F5’-CAGCTGTTCTCCTTCGGTTCT-3’  R5’-GAATGCTTCGCCATCACA3’ | 52˚C | 84˚C | 117 bp | [NM_001046172](http://www.ncbi.nlm.nih.gov/entrez/viewer.fcgi?db=nuccore&val=114051422) | NA^3^ |

^1^Additional primers and quantitative PCR conditions for the callipyge region of ovine chromosome 18 sequences and microarray target sequences have been published [18, 20, 47, 48].

^2^Percent similar identity of cloned sheep cDNA amplicon compared to bovine cDNA sequence.

^3^Sheep amplicon not sequenced due to lack of significance for quantitative PCR analysis.

^4^Designed directly from sheep sequence.
